# Stomach contents of long-finned pilot whales, *Globicephala melas* mass-stranded in Tasmania

**DOI:** 10.1371/journal.pone.0206747

**Published:** 2019-01-14

**Authors:** Isabel Beasley, Yves Cherel, Sue Robinson, Emma Betty, Rie Hagihara, Rosemary Gales

**Affiliations:** 1 Snubfin Dolphin Project, Colebrook, Tasmania, Australia; 2 College of Science and Engineering, James Cook University, Townsville, Australia; 3 Centre d’Etudes Biologiques de Chizé (CEBC), UMR 7372 du CNRS-Université de La Rochelle, Villiers-en-Bois, France; 4 Invasive Species Branch, Biosecurity Tasmania, Department of Primary Industries, Parks, Water and Environment, Tasmania, Australia; 5 Institute for Applied Ecology New Zealand, School of Science, Auckland University of Technology, Auckland, New Zealand; 6 Institute of Natural and Mathematical Sciences, College of Sciences, Massey University, Palmerston North, New Zealand; 7 Natural Values Conservation Branch, Department of Primary Industries, Parks, Water and Environment, Tasmania, Australia; University of Saint Andrews, UNITED KINGDOM

## Abstract

New data are reported from analyses of stomach contents from 114 long-finned pilot whales mass-stranded at four locations around Tasmania, Australia from 1992–2006. Identifiable prey remains were recovered from 84 (74%) individuals, with 30 (26%) individuals (17 females and 13 males) having empty stomachs. Prey remains comprised 966 identifiable lower beaks and 1244 upper beaks, belonging to 17 families (26 species) of cephalopods. Ommastrephidae spp. were the most important cephalopod prey accounting for 16.9% by number and 45.6% by reconstructed mass. *Lycoteuthis lorigera* was the next most important, followed by *Ancistrocheirus lesueurii*. Multivariate statistics identified significant differences in diet among the four stranding locations. Long-finned pilot whales foraging off Southern Australia appear to be targeting a diverse assemblage of prey (≥10 species dominated by cephalopods). This is compared to other similar studies from New Zealand and some locations in the Northern Hemisphere, where the diet has been reported to be primarily restricted to ≤3 species dominated by cephalopods. This study emphasises the importance of cephalopods as primary prey for Southern long-finned pilot whales and other marine vertebrates, and has increased our understanding of long-finned pilot whale diet in Southern Ocean waters.

## Introduction

Cephalopods comprise a major portion of the diets of many marine vertebrates (cetaceans, seals, birds and fish), and are a key trophic link in the Southern Ocean ecosystem [1–6]. Marine mammals spend their lives at sea and afford little opportunity for direct observation of feeding. Subsequently, indirect methods are often used in an attempt to reconstruct diet, such as analysis of stomach contents from stranded and by-caught individuals [7], and analysis of tissue lipid profiles [8–11] or stable isotopes [12–14].

Cetaceans frequently strand along the Tasmanian coastline, with published records since 1945 [15–18]. Between 1990 and 2008, a total of 336 stranding events occurred, totalling 2273 individuals. The most commonly stranded cetacean (by number of individuals) is the long-finned pilot whale, *Globicephala melas* (LFPW), where of 1568 individuals stranded (69% of all individuals during this time period), only 30 (8.9%) of the 336 stranding events were LFPWs. Although LFPWs strand in Tasmania throughout the year (except May and June), there is a distinct stranding peak during summer, from September to December (DPIPWE unpublished data). This apparent seasonality is also observed in LFPWs that strand along the New Zealand coastline [2, 3, 19, 20], and be as a result of long-distance migrations of LFPWs past Tasmania during summer, or reflective of seasonal changes in prey distribution from offshore areas onto the continental shelf and near-shore waters.

LFPWs occur in oceanic and coastal waters in temperate and subpolar zones [21]. In the Northern Hemisphere, they are found in the North Atlantic Ocean (including the Western Mediterranean and North Sea) north of 20°N [22]. In the Southern Hemisphere, they range in the Southern South Pacific, South Atlantic and mostly across the Southern Ocean as far south as the Antarctic Polar Front, sometimes to 68°S [21, 23]. The Southern Hemisphere subspecies (*G*. *m*. *edwardii*) are taxonomically and geographically separated from those in the Northern Hemisphere (*G*. *m*. *melas*) [22, 24, 25]. LFPWs occur in relatively stable, maternally based pods with a polygynous mating system [24]. The species’ strong social structure makes it particularly vulnerable to herding in drive fisheries, such as occurs off the Faroe Islands [26], and to mass-stranding events, such as occur on Cape Cod, Massachusetts, USA [27], Farewell Spit, New Zealand [28], and Tasmania, Australia [18]. Although LFPWs are currently considered circumglobal in the Southern Hemisphere, potential foraging differences may assist in elucidating distribution and potential stock structure differences, as well as the potential for fisheries interactions, which is fundamental knowledge for effective management of the species.

Diet studies have analysed the stomach contents obtained from LFPWs in many parts of the world [3, 4, 29–31], where in general, cephalopods are a main component of LFPW diet, although fish may also be important in some areas [32–34]. Previous reports of LFPW food habits have yielded three dietary patterns: (1) diverse diet (≥10 prey species) dominated by cephalopods [4, 29, 30]; (2) restricted diet (≤3 species) dominated by cephalopods [35, 36]; and (3) restricted diet (≤3 species) dominated by fish [34, 37, 38]. There is little known about the foraging behaviour of LFPWs in the Southern Hemisphere. Two LFPWs stranded on the Freycinet Peninsula on the East coast of Tasmania were found to have had a diverse diet (≥10 prey species) dominated by 14 cephalopod species [4]. *Sepioteuthis australis* was the most common cephalopod species in the diet of these animals (35.7% by number and 48.5% dry weight). Similar investigations into the diet of 14 LFPWs stranded in two events along the New Zealand coastline found a restricted diet (≤3 species) dominated by *Nototodarus* spp. and *Octopus maorum* [3, 20, 39]. The diet of Southern Hemisphere LFPWs has also been investigated in Chile [40], Argentina [41], southern Brazil [42], South Africa [43] and Antarctic/Sub-Antarctic [44]. In all studies cephalopods were found to constitute the main prey.

In this study, we examined the stomach contents of LFPWs mass stranded along the Tasmanian coastline. We characterise LFPW diet and compare the results to other global studies, particularly from the Southern Hemisphere. This study aims to assess what prey are important for LFPWs utilising Tasmanian waters, and subsequently consider the conservation implications of these dietary preferences.

## Materials and methods

### Sample collection

**Fig 1:**
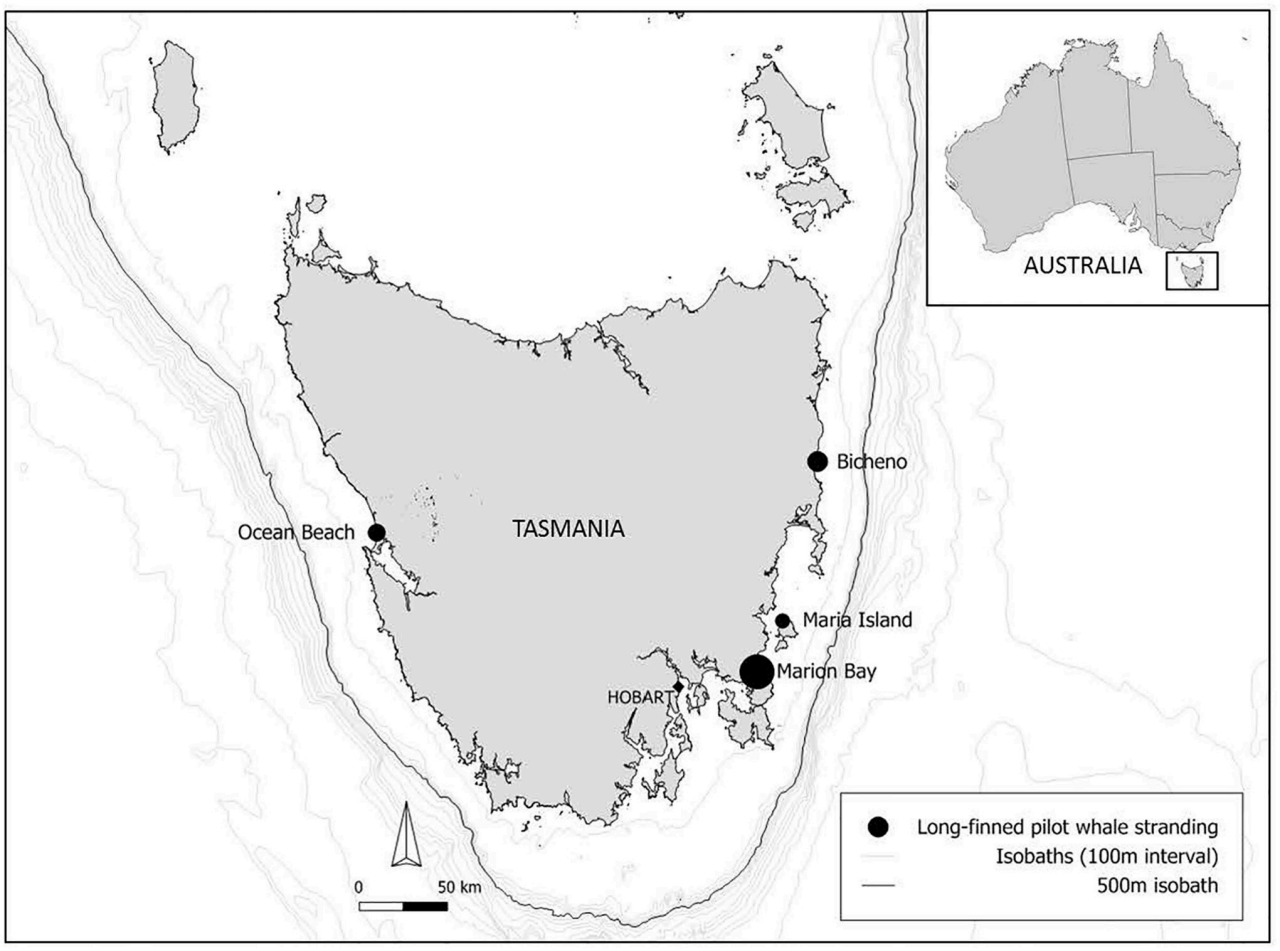
Location of LFPW mass-strandings around Tasmania from 1992 to 2006. The size of the location icon is representative of the number of individuals that stranded at that location (see Table 1): 24 individuals stranded at Bicheno (26/09/92), 41 at Maria Island (29/11/04), 161 at Marion Bay (25/10/05) and 27 at Ocean Beach (01/12/2006). At total of 24, 19, 49 and 22 stomach samples were available from these sites respectively.

Samples were collected from 114 LFPWs that did not survive mass stranding events along the Tasmanian coastline between September 1992 to December 2006 (Fig 1; Table 1).

**Table 1:** Summary of sex, body length and age (where available) of LFPWs for each stranding event from 1992 to 2006, with mean ± SD (range) (n = total individual included in sample size).

	Bicheno	Maria Island	Marion Bay	Ocean Beach
**Date of stranding**	26/09/92	29/11/04	25/10/05	01/12/2006
**Number of individuals stranded (alive and dead)**	24	41	161	27
**Number of dead individuals**	24	19	145	25
**Number of individuals with stomach contents**	24	19	49	22
**Total length of all dead individuals (m)**	—	4.7 ± 0.53 (4.1–5.9)	4.3 ± 0.71 (2.3–6.1)	4.2 ± 0.11 (2.0–5.6)
**- Females**	—	4.4 ± 0.13 (4.1–4.5) (n = 12)	4.3 ± 0.47 (2.7–4.8) (n = 38)	3.8 ± 0.89 (2.0–4.4) (n = 10)
**- Males**	—	5.2 ± 0.50 (4.2–5.9) (n = 7)	4.6 ± 0.12 (2.3–6.1) (n = 11)	4.6 ± 0.11 (2.1–5.6) (n = 12)
**Total length of dead individuals with some stomach contents (m)**	—	4.7 ± 0.51 (4.2–5.6) (n = 12)	4.3 ± 0.58 (2.7–5.7) (n = 43)	4.4 ± 0.80 (2.5–5.6) (n = 15)
**- Females**	—	4.4 ± 0.90 (4.2–4.5) (n = 8)	4.3 ± 0.49 (2.7–4.8) (n = 35)	3.9 ± 0.65 (3.8–4.4) (n = 8)
**- Males**	—	5.3 ± 0.42 (4.7–5.6) (n = 4)	4.5 ± 0.89 (3.3–5.7) (n = 8)	4.85 ± 0.69 (4.0–5.6) (n = 7)
Age estimates of dead individuals (years)*	—	—	17 ± 8.1 (2–32) (n = 32)	19 ± 12.4 (1–51) (n = 21)
**- Females**	—	—	17 ± 8.1 (2–32) (n = 32)	20 ± 13.6 (1–51) (n = 10)
**- Males**	—	—	15 ± 8.1 (2–23) (n = 10)	17 ± 11.6 (1–42) (n = 11)
**Age estimates of dead individuals with some stomach contents (years)**	—	—	17 ± 8.1 (2–32) (n = 36)	21 ± 11.1 (9–51) (n = 14)
**- Females**	—	—	18 ± 8.4 (2–32) (n = 29)	23.6 ± 12.93 13–51 (n = 8)
**- Males**	—	—	15 ± 7.1 (6–23) (n = 7)	17 ± 7.0 (9–27) (n = 6)

* Age estimated using growth layer patterns from teeth

Morphometric data, tissue samples and stomach contents were collected from each of the deceased whales (where logistically possible), following the protocols of Geraci and Lounsbury [45]. In the absence of life history data for Southern Hemisphere LFPWs, individuals were classified into age groups based on total length measurement, following Bloch et al. [46] and Desportes and Mouritsen [29] for Northern Hemisphere LFPWs from the Faroe Islands. These categories were: (1) newborn/calf (not fully weaned: male 1.78–2.20 m, female 1.74–1.90 m), (2) subadult (nutritionally independent, but sexually immature: male 2.20–4.80 m, female 2.00–3.75 m), or (3) adult (mature—male: >4.80 m, female >3.75 m). Sexual maturity is reported to be attained at an average age of eight years for females and 17 years for males [47, 48]; longevity is 35 to 45 years for males and can exceed 60 years for females [46]. It is acknowledged that these life history parameters may differ for Southern Hemisphere LFPWs. In addition, age estimates were available for some individuals using standard tooth aging methodologies [49], based on counts of the number of growth layers in a tooth taken from that animal (DPIPWE unpublished data).

### Laboratory analysis

Stomachs were excised on site and frozen until further analysis. Prior to analysis, the stomach contents were thawed, rinsed through a 1.0 mm sieve and sorted. When present, parasites were collected and preserved in 70% ethanol. Cephalopod remains were fixed in 5% buffered formalin solution, and then preserved in 70% ethanol. Cephalopod beaks were separated from other cephalopod hard part remains and sorted into upper and lower beaks. The lower beaks were identified to the lowest possible taxonomic level using Xavier and Cherel [50] and with the aid of cephalopod reference collections held at the Centre d’Etudes Biologiques de Chizé, France (specimens identified by Drs. Yves Cherel and Jose Xavier); Auckland University of Technology (specimens identified by Drs. Steve O’Shea and Emma Betty); and the Institute for Marine and Antarctic Studies, University of Tasmania (specimens identified by Dr. Karen Evans). Identification of the teleost otoliths was carried out using Furlani *et al*. [51].

### Sample analysis

To estimate the original size of the cephalopod prey, lower rostral lengths (LRLs) for decapods and lower hood lengths (LHLs) for octopods were measured with digital callipers to the nearest 0.1 mm, or (for very small beaks) with a micrometer under a binocular microscope (n = 2). Regression equations were used as constructed by Clarke [52], Rodhouse and Yeatman [53], Lu and Ickeringill [54], Beatson and O’Shea [55], Horstkotte [56] and Xavier and Cherel [50] (S1 Table). The relative importance of prey items was quantified by: (1) frequency of occurrence (FO), defined as the proportion of stomachs that contained a particular prey species, regardless of mass or abundance; (2) proportion of numerical abundance (%Num), the percentage of the total number of prey items recovered from all stomachs represented by a particular prey category; (3) proportion of reconstructed prey mass (%Mass), the percentage of reconstructed mass of prey recovered from all stomachs represented by a particular prey category; and (4) index of relative importance (IRI), which combines the above three methods and is calculated using the formula: IRI = FO x (%Num + %Mass) (*sensu* [57]). The reconstructed mass of prey for each stranding was obtained by totalling the reconstructed prey mass for each individual from that stranding. We acknowledge that there are several potential biases of using the reconstructed prey mass (RPM) where, (1) the final RPM may be an underestimation since broken, upper only or unidentified beaks did not contribute to the total estimate prey mass [20], (2) the final RPM may be an overestimation as a result of the potential for accumulation of prey items over time [58], and (3) many regressions are based on small sample sizes and do not include a comprehensive coverage of size distributions, which results in an inherent uncertainty in the mass calculated [5].

### Statistical analysis

Classification Trees (CTs) were used to identify prey species that distinguished LFPWs stranded between the four locations and between sexes, using R package rpart [59]. CTs use tree-building algorithms which examine each response variable (prey species), one at a time, selects one variable that minimizes the classification error, splits the predictor into two groups, decides when a branch is terminal (stopping rules) and predicts multiple classes or a binary class at end points [59]. The CT was pruned at a node that minimized the overall classification error. Due to missing values, five prey species were excluded from the analysis. Twenty-one prey species were subsequently used in the analysis. The prey species that were identified as important in the CT were then examined in Kruskal-Wallis rank sum test for significance testing. Age class was not examined statistically as adults dominated samples collected from Marion Bay and Ocean Beach, sub-adults were mostly from Ocean Beach, and there was no information on age class from Bicheno.

## Results

A total of 253 LFPWs mass-stranded at four locations around Tasmania from 1992–2008 (Bicheno, Maria Island, Marion Bay and Ocean Beach) (Fig 1). Of these LFPWs, 213 subsequently died, while 40 were released alive. Stomach contents were collected from 114 LFPWs, representing 54% of all deceased whales that mass stranded and died, in these four events. Out of the 114 individuals, prey remains were recovered from 84 (74%) individuals, with 30 (26%) individuals (17 females and 13 males) having empty stomachs (Table 2). The one calf (Ocean Beach), and three juveniles (two from Ocean Beach and one from Marion Bay) did not have any recognisable stomach contents.

**Table 2:** Summary of the percentage of LFPW stomachs with prey contents, empty stomachs and parasites only in stomach. For stomachs with prey contents, the percentage of identifiable cephalopod beaks, and upper or broken beaks is also shown.

Dietary group	n	Prey Contents	Empty Stomachs	Parasites Only	Identifiable Lower Cephalopods Beaks	Upper or Broken Beaks Only
**All**	**114**	**84 (74%)**	**9 (18%)**	**9 (8%)**	**69 (82%)**	**15 (18%)**
Bicheno	24	22 (92%)	2 (8%)	0	20 (91%)	2 (9%)
Maria Island	19	12 (63%)	7 (37%)	0	8 (67%)	4 (33%)
Marion Bay	49	35 (71%)	6 (12%)	8 (17%)	29 (83%)	6 (17%)
Ocean Beach	22	15 (68%)	6 (27%)	1 (5%)	12 (80%)	3 (20%)
Females (total)	78	61 (78%)	9 (12%)	8 (10%)	49 (80%)	15 (20%)
Males (total)	36	23 (64%)	12 (33%)	1 (3%)	20 (87%)	3 (13%)

Out of the 84 stomachs with prey contents, 69 (82%) contained identifiable lower cephalopod beaks. The remaining stomach samples contained unidentified/broken lower beaks and upper beaks, and/or squid eye lenses only (Table 3). In addition to cephalopod beaks, other cephalopod remains recovered included complete and partial eye lenses and sucker rings and hooks. No buccal masses were present. Out of the 114 stomachs recovered, 43% of stomachs contained intestinal nematodes.

**Table 3:** Summary of the percentage of cephalopod, cephalopod eyeball, fish and nematode remains found in LFPWs, separated by stranding event, and sex.

Dietary group	n	% Containing Cephalopods	% Containing Eyeballs	% Containing Fish	% Containing Nematodes
**All**	**114**	**72.8**	**28.0**	**0.9**	**43.0**
**Bicheno**	24	87.5	83.3	0.0	20.8
**Maria Island**	19	38.7	9.7	3.2	25.8
**Marion Bay**	49	71.4	10.2	0.0	55.1
**Ocean Beach**	22	68.2	18.2	0.0	40.9
**Females (total)**	78	76.9	29.5	0.0	50.0
**Males (total)**	36	72.2	25.0	2.8	27.8

### Cephalopod species composition

Cephalopod prey remains comprised 966 identifiable lower beaks (an additional 65 lower beaks were broken and unidentifiable) and 1244 upper beaks, representing species from two cephalopod orders; Octopoda (Octopodiformes), and Teuthida (Decapodiformes). Octopoda were represented by two species from two families (*O*. *maorum* and *Ocythoe turberculata)* and Teuthida by 24 species from 15 families (Table 4). An average of 12 ± 5.5 (4–16) taxa were recovered from each stranding event, with a minimum of four cephalopod species present from Maria Island samples, and a maximum of 16 species from Bicheno samples. Only two cephalopod species: *Lycoteuthis lorigera* and Ommastrephidae spp., were present in the stomach of at least one whale from all four stranding locations.

**Table 4:** Measured lower rostral lengths, and calculated mantle lengths and wet weight ± SD (range) of cephalopod species in the stomachs of LFPW from Tasmania.

Species (n)	LRL (mm)	ML (mm)	Wet mass (g)
**Ancistrocheiridae**			
*Ancistrocheirus lesueurii* (n = 101)	7.8 ± 1.1 (3.3–9.8)	360 ± 45 (177–440)	1346 ± 470 (60–2763)
**Architeuthidae**			
*Architeuthis dux* (n = 3)	8.5 ± 0.9 (7.8–9.5)	448 + 52 (406–507)	3173 + 1557 (2015–4943)
**Brachioteuthidae**			
*Brachioteuthis linkovskyi* (n = 8)	3.5 ± 0.4 (3.1–4.3)	87 + 8 (79–102)	10 ± 2 (9–13)
**Chiroteuthidae**			
*Chiroteuthis capensis* (n = 13)	4.8 ± 0.4 (4.2–5.4)	140 ± 34 (101–209)	55 ± 13 (38–74)
*Chiroteuthis* sp. F (Imber) (n = 1)	5.4	143	74
*Chiroteuthis veranyi* (n = 13)	6.1 ± 1.2 (4.3–7.6)	161 ± 29 (117–196)	113 ± 54 (41–184)
**Cranchiidae**			
*Galiteuthis* sp. (n = 2)	4.4 ± 1.09 (3.6–5.2)	217 ± 9 (211–223)	70 ± 39 (42–97)
*Megalocranchia* sp. (n = 13)	8.8 ± 0.96 (7.6–10.4)	491 ± 60 (402–627)	344 ± 107 (230–531)
*Teuthowenia pellucida* (n = 110)	4.3 ± 0.35 (2.9–5.0)	151 ± 10 (110–172)	35 ± 5 (16–47)
**Enoploteuthidae**			
*Enoploteuthis* sp. (n = 8)	3.4 ± 0.32 (3.2–4.0)	84 ± 9 (76–100)	24 ± 6 (19–35)
**Histioteuthidae**			
*Histioteuthis atlantica* (n = 82)	4.3 ± 0.44 (2.9–5.6)	96 ± 11 (66–133)	139 ± 47 (47–334)
*Histioteuthis macrohista* (n = 1)	3.28	53	69
*Histioteuthis miranda* (n = 3)	2.5 ± 0.16 (2.3–2.6)	59 ± 4 (54–62)	36 ± 7 (29–42)
**Loliginidae**			
*Sepioteuthis australis* (n = 5)	5.0 ± 0.43 (4.4–5.5)	321 ± 29 (279–351)	262 ± 60 (177–327)
**Lycoteuthidae**			
*Lycoteuthis lorigera* (n = 397)	5.1 ± 0.51 (3.2–5.9)	163 ± 20 (76–192)	190 ± 49 (44–286)
**Mastigoteuthidae**			
?*Mastigoteuthis* A (Clarke) (n = 1)	5.9	131	2
**Neoteuthidae**			
*Nototeuthis dimegacotyle* (n = 3)	3.5 ± 0.12 (3.4–3.6)	na	na
**Octopoteuthidae**			
*Octopoteuthis* sp.	13.7 ± 1.88 (9.6–15.3)	237 ± 33 (165–265)	513 ± 142 (217–645)
**Ommastrephidae**			
*Martialia hyadesi* (n = 2)	4.9 ± 0.78 (4.4–5.5)	246 ± 23 (263–230)	275 ± 87 (213–336)
Ommastrephidae sp. (n = 163)	10.2 ± 2.32 (4.4–15.0)	406 ± 88 (226–742)	1552 ± 852 (149–3829)
**Onychoteuthidae**			
*Onychoteuthis banksii* complex (n = 2)	2.9 ± 0.24 (2.7–3.0)	96 ± 23 (91–102)	19 ± 4 (16–22)
*Onykia robsoni* (n = 1)	8.44	622	3175
*Notonykia africanae* (n = 2)	3.5 ± 0.81 (2.9–4.1)	na	na
**Pholidoteuthidae**			
*Pholidoteuthis massyae* (n = 19)	10.8 ± 1.81 (6.2–13.6)	454 ± 74 (267–571)	2368 ± 1007 (470–4292)
**Octopodidae**			
*Octopus maorum* (n = 7)	5.1 ± 0.53 (4.4–5.8)	106 ± 15 (84–126)	512 ± 130 (339–692)
**Ocythoidae**			
*Ocythoe turberculata* (n = 3)	5.9 + 1.09 (5.0–7.1)	37 + 6 (31–44)	32 + 15 (20–49)

Ommastrephidae spp. included several species whose beaks cannot be easily differentiated by their morphology, namely *Nototodarus gouldii*, *Ommastrephes bartrami* and *Todarodes* sp. (including *T*. *filippovae*). Allometric equations were therefore used at the family level to estimate their body length and mass.

### Cephalopod prey size and total biomass consumed

The reconstructed prey mass was highest for an adult male whale from Bicheno, with an estimated prey mass of 42.6 kg. The average LRL, Mantle Length (ML) and Biomass (BM) for each squid species are summarised in Table 5. The smallest squid recovered (by BM) was an *O*. *turberculata* with an estimated BM of 19.9 g (ML = 31.4 mm). The largest squid recovered was *Architeuthis dux* (giant squid), with an estimated BM of 4943 g (ML = 507 mm) (Table 4).

**Table 5:** A summary of the species composition and relative importance of prey items (FO, %Num, %Mass and IRI), for all four strandings combined. ‘Count’ is the number of whale stomachs that the cephalopod species were recovered from.

Species	Total N	Count	FO	%Num	BM (g)	%Mass	IRI
**Ancistrocheiridae**							
*Ancistrocheirus lesueurii*	101	24	0.3	10.5	135983	24.5	12.2
**Arthiteuthidae**							
*Architeuthis dux*	3	3	0.0	0.3	9520	1.7	0.1
**Brachioteuthidae**							
*Brachioteuthis linkovskyi*	8	7	0.1	0.8	81	0.0	0.1
**Chiroteuthidae**							
*Chiroteuthis capensis*	13	6	0.1	1.3	723	0.1	0.1
*Chiroteuthis* sp. F (Imber)	1	1	0.0	0.1	74	0.0	0.0
*Chiroteuthis veranyi*	12	6	0.1	1.2	1473	0.3	0.1
**Cranchiidae**							
*Galiteuthis* sp.	2	2	0.0	0.2	139	0.0	0.0
*Megalocranchia* sp.	13	8	0.1	1.3	4474	0.8	0.2
*Teuthowenia pellucida*	105	22	0.3	10.9	3648	0.7	3.7
**Enoploteuthidae**							
*Enoploteuthis* sp.	8	6	0.1	0.8	189	0.0	0.1
**Histioteuthidae**							
*Histioteuthis atlantica*	82	19	0.3	8.5	11427	2.1	2.9
*Histioteuthis macrohista*	1	1	0.0	0.1	77	0.0	0.0
*Histioteuthis miranda*	3	3	0.0	0.3	167	0.0	0.0
**Loliginidae**							
*Sepioteuthis australis*	5	3	0.0	0.5	1308	0.2	0.0
**Lycoteuthidae**							
*Lycoteuthis lorigera*	397	44	0.6	41.1	75465	13.6	34.9
**Mastigoteuthidae**							
?*Mastigoteuthis* A (Clarke)	1	1	0.0	0.1	2	0.0	0.0
**Neoteuthidae**							
Nototeuthis dimegacotyle*	3	3	0.0	0.3	na	na	na
**Octopoteuthidae**							
*Octopoteuthis* sp.	9	6	0.1	0.9	4616	0.8	0.2
**Ommastrephidae**							
*Martialia hyadesi*	2	1	0.0	0.2	549	0.1	0.0
Ommastrephidae sp.	163	43	0.6	16.9	252996	45.6	38.9
**Onychoteuthidae**							
*Onychoteuthis banksia* complex	2	2	0.0	0.2	37	0.0	0.0
*Onykia robsoni*	1	1	0.0	0.1	3175	0.6	0.0
Notonykia africanae*	2	2	0.0	0.2	na	na	na
**Pholidoteuthidae**							
*Pholidoteuthis massyae*	19	13	0.2	2.0	44994	8.1	1.9
**Octopodidae**							
*Octopus maorum*	7	3	0.0	0.7	3582	0.6	0.1
**Ocythoidae**							
*Ocythoe turberculata*	3	3	0.0	0.3	96	0.0	0.0
**Total Lower Cephalopod Beaks**	**966**			**100.0**	**554792**	**100.0**	

* regression equations not available

**Fig 2:**
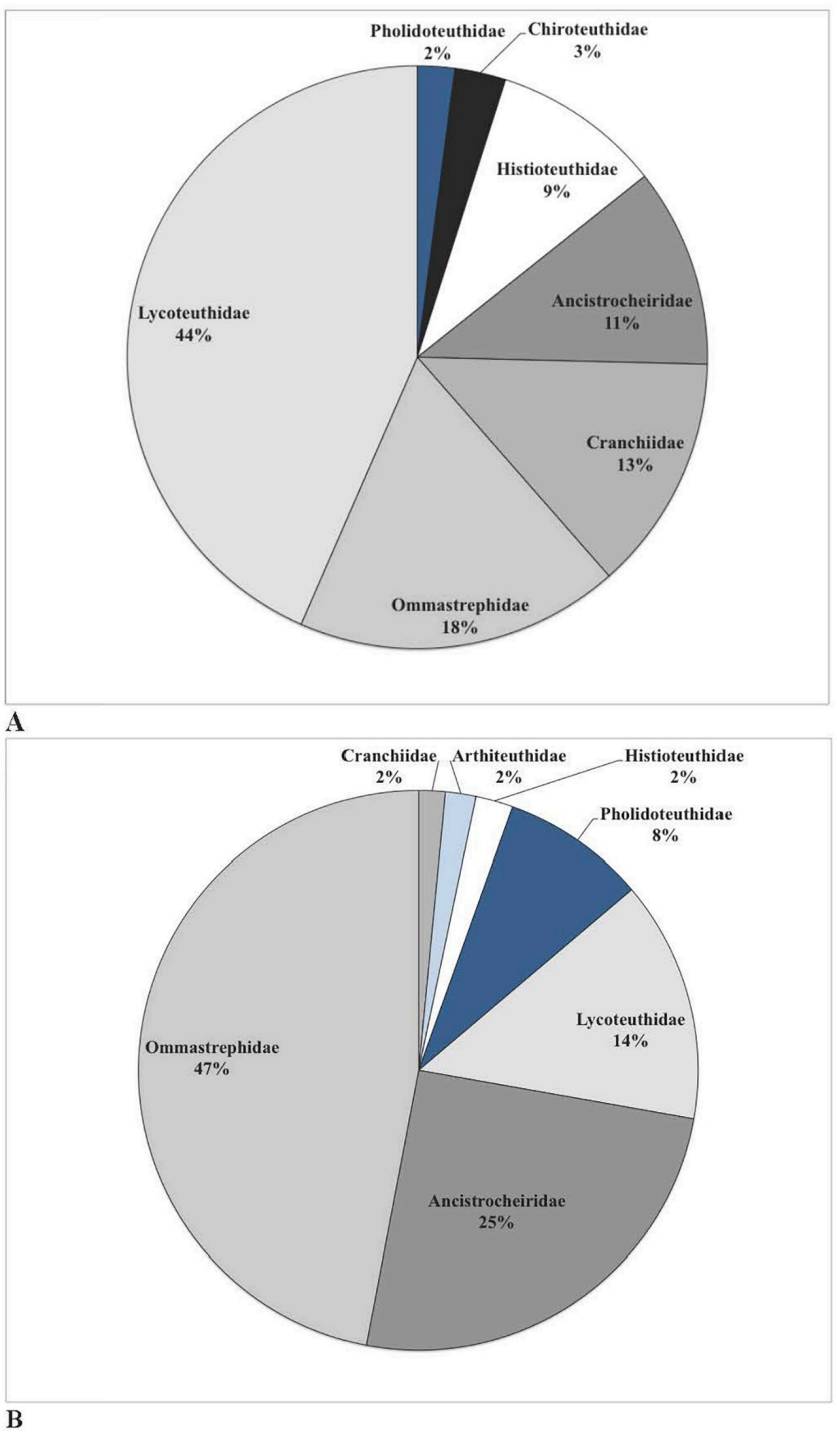
(A) Percent numerical abundance (%Num) and (B) percent reconstructed prey mass (%BM) of cephalopod genera found in the diet of LFPWs stranded along the Tasmanian coastline from 1992 to 2006. Species where the %Num and %BM were <1% are not included.

**Fig 3:**
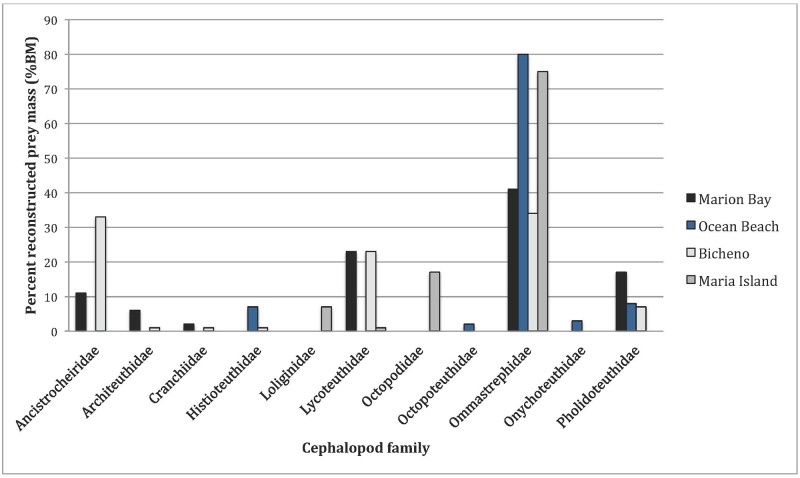
Percent reconstructed prey mass (%BM) of cephalopod families found in the diet of LFPWs stranded along the Tasmanian coastline from the four stranding locations. Species where the %BM are <1% are not included.

A summary of the species composition and relative importance of prey items (FO, %Num, %Mass and IRI) is presented in Table 5. For all strandings combined, Ommastrephidae spp. were the most important cephalopod prey accounting for 16.9% by number and 45.6% by reconstructed mass. *L*. *lorigera* was the next most important (41.1% by number and 13.6% by reconstructed mass), followed by *Ancistrocheirus lesueurii* (10.5% by number and 24.5% by reconstructed mass; Fig 2). The percent reconstructed prey mass for the four stranding locations are shown in Fig 3. As discussed above, it is important to highlight that the Ommastrephidae spp. grouping included at least three different species, which would have upwardly biased the relative importance of this category when compared to other single species categories.

### Variations in cephalopod consumption

#### Sex variation

Among the 69 individuals with identifiable prey contents, no differences in total reconstructed prey mass were observed in diet between males (n = 19) and females (n = 55) (Kruskal-Wallis rank sum test: Chisq = 0.76, df = 1, p = 0.38). Females consumed a reconstructed prey mass average per individual of 5.4 kg ± 8.8 (0.1–38.2 kg), while males consumed a similar reconstructed prey mass average of 4.1 kg ± 7.5 (0.1–30.6 kg). For both sexes, Ommastrephidae spp. was the most important cephalopod prey, followed by *L*. *lorigera* and *A*. *lesueurii* (Table 6). These three species together comprised 75% and 86% reconstructed mass of prey ingested, for males and females, respectively. These results were supported by the CT analysis, where no nodes were identified in the classification tree. Because of a lack of diet difference between males and females, all subsequent results were pooled to include both males and females.

**Table 6:** Summary of the three most important cephalopod species recovered for each sex.

Sex	n	IRI	Species	%Num	%Mass	Total Mass (kg)
**Female**	50	30.3	Ommastrephidae sp.	17.4	47.9	336.6
		28.64	*L*. *lorigera*	43.2	14.9	
		9.08	*A*. *lesueurii*	9.7	23.2	
		**Total %Num and %Mass**		**70.3**	**86.1**	
**Male**	19	9.54	Ommastrephidae sp.	15.4	44.5	111.1
		6.81	*L*. *lorigera*	35.4	11.6	
		2.29	*A*. *lesueurii*	12.3	19.3	
		**Total %Num and %Mass**		**63.1**	**75.4**	

#### Age-class variation

When separating individuals according to age class, no total length information was available from individuals at the Bicheno stranding. Therefore the age-classes of these 20 individuals were unknown and excluded from further age-class variation analysis. Of the remaining 49 individuals with recognisable prey contents (subadults and adults), 35 (71%) individuals were aged by conventional aging techniques using growth layer groups (GLGs), and the remaining 14 (29%) individuals were assigned an age class based only on total length [46]. Age-class variation analysis therefore consisted of 6 (12%) subadults (2 females and 4 males) from Marion Bay and Ocean Beach, and 43 (88%) adults (33 females and 10 males), from all stranding sites except Bicheno (Table 7). Because of the low number of subadults from Marion Bay (n = 1), age-class comparisons could only be undertaken for Ocean Beach.

**Table 7:** Summary of the three most important cephalopod species recovered for each age class at Ocean Beach.

Age-class	Location	n	# prey species	IRI	Species	%Num	%Mass
**Subadult**	Ocean Beach	5	12	7.71	Ommastrephidae sp.	38.4	50.3
				2.95	*H*. *atlantica*	38.4	2.3
				0.53	*P*. *massyae*	4.7	13.5
		**Total %Num and %Mass**				**81.5**	**66.1**
**Adult**	Ocean Beach	7	12	6.3	Ommastrephidae sp.	26.9	81.7
				2.5	*H*. *atlantica*	38.5	5.3
				0.2	*Octopeteuthis sp*.	3.9	2.9
		**Total %Num and %Mass**				**69.3**	89.9

There were no apparent differences in the diversity of cephalopod species consumed by LFPWs stranded at Ocean Beach, with 12 species consumed by both adults and subadults. According to the IRIs, the most important cephalopod species for adults and subadults were Ommastrephidae sp., followed by *H*. *atlantica* (Table 7). The small sample size of subadults precluded any reliable multivariate statistical analysis; however the total reconstructed weight was not significantly different between adults and subadults from Ocean Beach (Kruskal Wallis rank sum test: Chisq = 1.27, df = 1, p = 0.26).

#### Size of cephalopods

**Fig 4:**
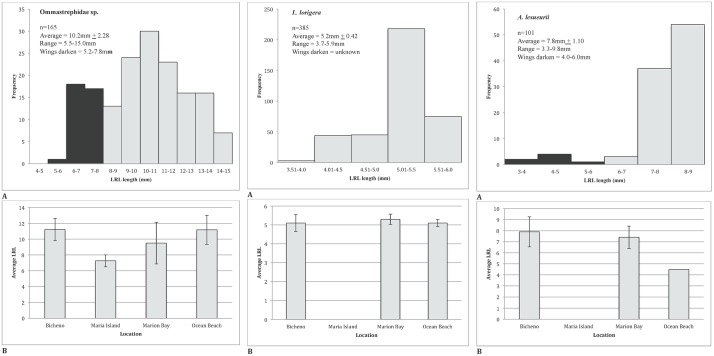
(A) Frequency histograms of the lower rostral lengths (LRLs) of the three most important squid species in the diet of LFPWs stranded in Tasmanian waters: *Ommastrephidae spp*., *Lycoteuthis lorigera and Ancistrocheirus lesueurii*, for all four stranding locations combined. Shaded areas show the young beaks with undarkened wings (unknown LRL for *L*. *lorigera*). (B) Average LRL (±SD) of the three most important squid species in the diet of LFPWs stranded in Tasmanian waters, separated by stranding location.

Comparison of size frequency distributions of the three most important cephalopod species: Ommastrephidae spp., *L*. *lorigera and A*. *lesueurii* showed a consistent preference for adults. Average LRLs were 10.2 mm ±2.28 (5.5–15.0), 5.2 mm +0.42 (3.7–5.9) and 7.8 mm ±1.10 (3.3–9.8), respectively (Fig 3), with wings darkening at a LRL of 5.2–7.8 mm, range unknown and 4.0–6.0 mm, respectively [60] (Fig 4).

#### Stranding location variation

All stranding sites varied in the diversity and proportion of cephalopod species present, as well as the most important cephalopod species (Table 8). According to the resulting IRIs, Ommastrephidae spp. was the most important cephalopod prey item for Maria Island (East coast) and Ocean Beach samples (West coast), while *L*. *lorigera* was the most important for Marion Bay and Bicheno samples (both East coast). The greatest total reconstructed prey mass was from whales in Bicheno (mean = 13.4 kg, SD = 12.2 kg), followed by Ocean Beach (mean = 5.1 kg, SD = 6.3 kg), Marion Bay (mean = 1.7 kg, SD = 3.8 kg) and Maria Island (mean = 1.1 kg, SD = 3.0 kg) (Table 9).

**Table 8:** Summary of the three most important cephalopod species recovered from each site. Data from Gales et al. (1992) are also presented.

Location	Coastline	Month	Year	n stomachs	n species present	Important three	% Num	% Mass	Total Mass
Bicheno	East Coast	September	1992	**22**	**16**	*L*. *lorigera*	46.2	22.1	88.1
						*A*. *lesueurii*	14.7	32.2	
						Ommastrephidae sp.	10.1	33.8	
Maria Island	East Coast	November	2004	8	4	Ommastrephidae sp.	62.8	74.2	97.8
						*O*. *maorum*	16.3	17.3	
						*S*. *australis*	11.6	6.3	
Marion Bay	South East Coast	October	2005	29	13	*L*. *lorigera*	61.9	27.7	89.8
						Ommastrephidae sp.	16.3	48.7	
						*A*. *lesueurii*	5.0	13.4	
Ocean Beach	West Coast	December	2006	12	15	Ommastrephidae sp.	34.1	78.2	92.2
						*H*. *atlantica*	38.4	6.6	
						*P*. *massyae*	3.6	7.4	
Freycinet Peninsula	East Coast (Gales et al. 1992)	July	1986	2	14	*S*. *australis*	35.7	43.4	125.8
						*N*. *gouldi*	23.6	30.1	
						*Sepia apama*	12.1	17.9	

**Table 9:** Prey species and mean reconstructed mass of prey recovered from stomachs of LFPWs stranded in Bicheno, Maria Island, Marion Bay and Ocean Beach. The plus sign (+) indicates the presence of a prey species in the stomach of at least one whale.

	Location, Month and Year			
	Bicheno	Maria Island	Marion Bay	Ocean Beach
Species	Sept 1996	Nov 2008	Oct 2009	Dec 2010
*Ancistrocheirus lesueurii*	**+**		**+**	**+**
*Architeuthis dux*	**+**		**+**	
*Brachioteuthis linkovskyi*	**+**			**+**
*Chiroteuthis capensis*	**+**			
*Chirotethis* sp. F (Imber)	**+**			
*Chiroteuthis veranyi*	**+**		**+**	**+**
*Galiteuthis* sp.			**+**	**+**
*Megalocranchia* sp.	**+**		**+**	**+**
*Teuthowenia pellucida*	**+**		**+**	**+**
*Enoploteuthis* sp.	**+**		**+**	
*Histioteuthis atlantica*	**+**			**+**
*Histioteuthis macrohista*				**+**
*Histioteuthis miranda*				**+**
*Sepioteuthis australis*		**+**		
*Lycoteuthis lorigera*	**+**		**+**	**+**
?*Mastigoteuthis* A (Clarke)			**+**	
*Nototeuthis dimegactyle*	**+**			
*Octopoteuthis* sp.	**+**			**+**
*Martialia hyadesi*			**+**	
Ommastrephidae sp.	**+**	**+**	**+**	**+**
*Onychoteuthis banksii*			**+**	
*Notonykia africanae*	**+**			
*Onykia robsoni*				**+**
*Pholidoteuthis massyae*	**+**		**+**	**+**
*Octopus maorum*		**+**		
*Ocythoe turberculata*				**+**
**Number of individual whales**	**24**	**19**	**49**	**22**
**Mean and SD reconstructed prey mass (kg)**	**13.4 (12.2)**	**1.0 (3.0)**	**1.7 (3.8)**	**5.1 (6.3)**
**Stomach contents**	**Cephalopod beaks and eye lenses, empty**	**Cephalopod beaks and eye lenses, otoliths, parasites, empty**	**Cephalpod beaks and eye lenses, parasites, empty**	**Cephalpod beaks and eye lenses, parasites, empty**

**Fig 5:**
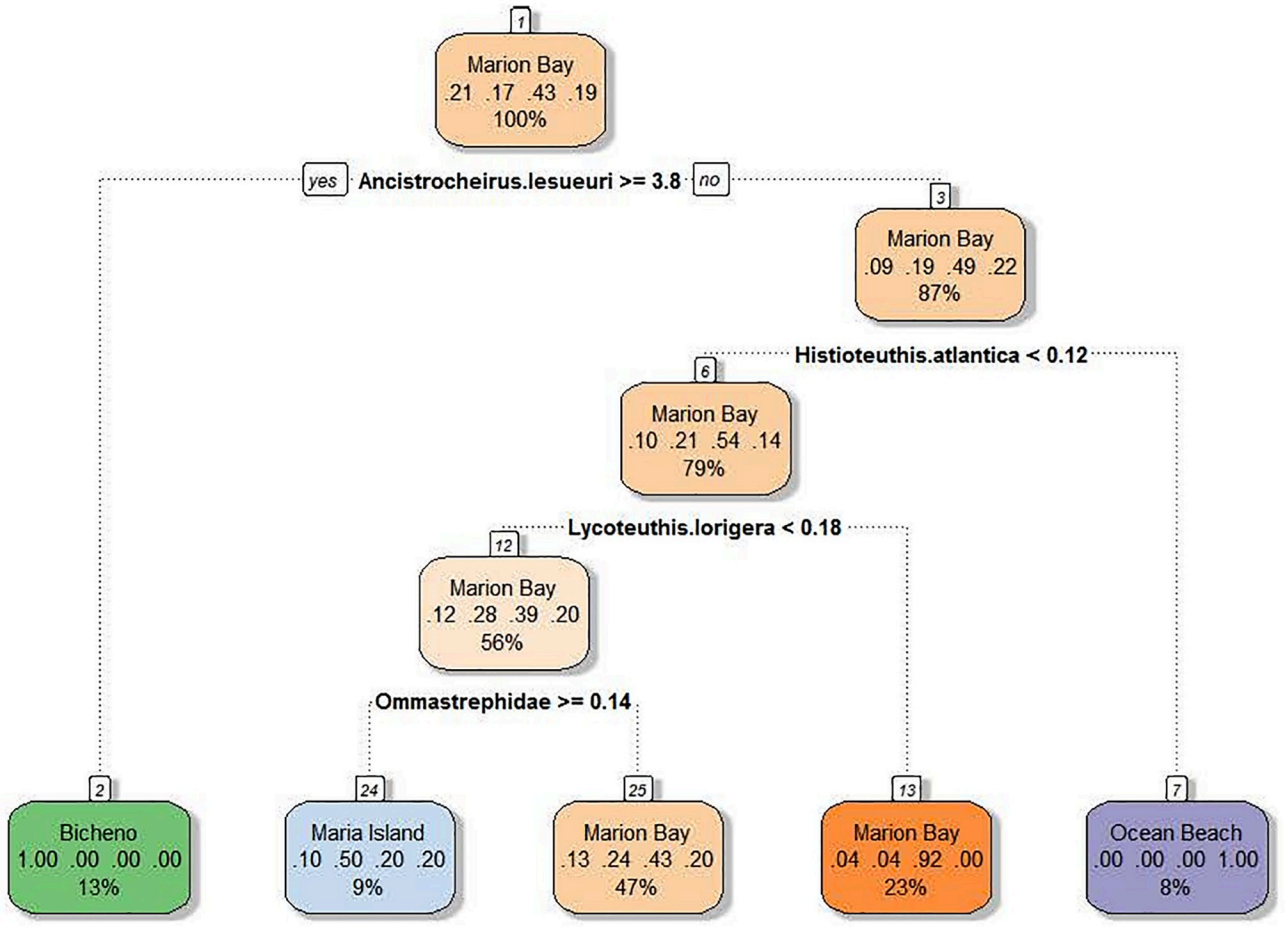
The pruned classification tree which splits prey items consumed by LFPWs stranded in four locations.

A CT comparing locations resulted in the decision tree showing four nodes, however with a high prediction error of 65%, probably due to a large number of zeros (Fig 5). The CT split was confirmed by subsequent univariate analysis. LFPWs stranded at Bicheno had significantly higher biomass of *A lesueuri* than the other three locations (Kruskal Wallis rank sum test: Chisq = 54.45, df = 1, p<0.0001). LFPWs stranded at Ocean Beach had significantly higher biomass of *Histioteuthis atlantica* than Marion Bay and Maria Island (Kruskal Wallis rank sum test: Chisq = 30.45, df = 1, p<0.0001). Although the CT node separated the Marion Bay stranded from the Maria Island stranding based on Ommastrephidae spp. biomass, further statistical analysis showed no significant difference (Kruskal Wallis rank sum test: Chisq = 0.02, df = 1, p = 0.88), thereby indicating that there was no difference in the prey composition from LFPWs stranded in Marion Bay and Maria Island.

The greatest proportion of cephalopod beaks by numerical abundance (65%) were recovered from individuals stranded at Bicheno, followed by Ocean Beach, Marion Bay and Maria Island. A total of 16 cephalopod species (62% of the total number of cephalopod species recovered) were recovered Bicheno samples, with four species (*Chiroteuthis capensis*, *C*. sp. F (Imber), *Nototeuthis dimegacotyle* and *Notonykia africanae*) only found at Bicheno and not recovered from other sites. Fifteen cephalopod species (58%) were recovered from Ocean Beach samples, amongst which were four cephalopod species that were not recovered at other sites: *H*. *miranda*, *H*. *macrohista*, *O*. *turberculata* and *Onykia robsoni*. Thirteen cephalopod species (50%) were recovered from Marion Bay samples, including *Martialia hyadesi* (a Subantarctic squid species), which was not recovered from any other stranding site. Only four cephalopod species were recovered from Maria Island samples, with two neritic cephalopod species, *S*. *australis* and *O*. *maorum*, being recovered that were not recorded from other sites.

### Correlation between LFPW body size and the three most important cephalopod prey

#### Location correlations

There was an overall negative correlation between LFPW body size and Ommastrephidae spp. average LRL (i.e. the larger the LFPW body size, the smaller the prey size consumed: Pearson’s correlation coefficient = -0.422: t = -2.324, df = 25, p = 0.028) (S1 File). When comparing stranding locations separately, a negative correlation was found between LFPW body size and Ommastrephidae spp. average LRL in Marion Bay (Spearman’s rank correlation coefficient = -0.72: S = 492, p = 0.011) but not in Maria Island (Spearman’s rank correlation coefficient = 0.10: S = 18, p = 0.95) or Ocean Beach (Spearman’s rank correlation coefficient = -0.21: S = 200, p = 0.554) (S1 File). No total length information was available for the Bicheno stranding, so Bicheno is not discussed further within these comparisons.

No correlation was found between LFPW body size and *L*. *lorigera* average LRL in Marion Bay (Spearman’s rank correlation coefficient = -0.357: S = 2747, p = 0.09) (S1 File). Correlation was not estimated for other locations because Maria Island had only one data point and Ocean Beach only two data points.

No correlation was found between LFPW body size and *A*. *lesueuri* average LRL in Marion Bay (Spearman’s rank correlation coefficient = -0.543: S = 54, p = 0.297) (S1 File). Correlation was not estimated for other locations because Maria Island had no data points and only one data point for Ocean Beach.

#### Sex correlations

For all sexes combined, there was a small negative correlation between LFPW body size and Ommastrephidae spp. average LRL (S1 File). For female LFPWs, their body size was negatively correlated with Ommastrephidae spp. average LRL (Spearman’s rank correlation coefficient = -0.65: S = 1886, p = 0.002) but not for male LFPWs (Spearman’s rank correlation coefficient = -0.62: S = 136, p = 0.10) (S1 File).

No correlation between whale body size and *L*. *lorigera* average LRL in both females (Spearman’s rank correlation coefficient = -0.208: S = 1606, p = 0.38) and males Spearman’s rank correlation coefficient = -0.20: S = 42, p = 0.71)(S1 File).

No correlation between LFPW body size and *A*. *lesueuri* average LRL in females (Spearman’s rank correlation coefficient = -0.543: S = 54, p = 0.297) (S1 File). Correlation was not estimated for males because there was only one data point.

### Non-cephalopod species composition

From 114 stomachs, only three otoliths were recovered from an adult male stranded at Maria Island on 30 November 2004 (TMAG Number = A2103). Two otoliths (from different individuals) were from the red cod, *Pseudophycis bachus* a temperate fish species which reaches 800 mm in length, and is distributed from New South Wales south to Tasmania, primarily in shallow sandy areas through to 375 m depth. The otolith lengths were 11 mm and 12 mm, indicating that the fish consumed were 351 mm and 383 mm, respectively [51]. The third otolith was too eroded to permit species identification.

Nematode parasites (of unknown species) were found in 49 stomachs (43%). The high prevalence of stomach nematodes from all stranding events is not discussed further within this paper; however it may be important for future investigation into factors causing LFPWs to strand [61, 62]. Similarly high numbers of intestinal nematodes were recovered from sperm whales stranded in Tasmanian waters in February 1998 [5, 63].

## Discussion

Of the 17 cephalopod families (26 species) identified in this study, the three most important cephalopod taxa in the diet of LFPW from Tasmanian waters are Ommastrephidae spp., *L*. *lorigera* and *A*. *lesueurii*. Additionally, *S*. *australis*/*O*. *maorum*, and *H*. *atlantica*/*P*. *massyae* were considered important prey for LFPWs recovered from Maria Island and Ocean Beach, respectively. A total of 226 cephalopod species have been recorded from Australian waters [64], therefore these 26 species represent only a small portion of species potentially available for consumption by LFPW.

All cephalopod species recovered have wide distributions outside Tasmanian waters, with some species not being found in Tasmanian waters (i.e. *M*. *hyadesi*). Therefore, there is inconclusive evidence to determine whether the whales were foraging in Tasmanian waters before they stranded. However, there are indications that some LFPWs may have been feeding in Tasmanian waters based on the presence of non-digested cephalopod remains (i.e. complete and partial eye lenses and sucker rings and hooks) in 28% of stomachs, and the presence of the near-shore cephalopod species *S*. *australis* and *O*. *maorum* in Maria Island/Ocean Beach and Maria Island stranding events, respectively.

### Variation by location, sex and age-class

Variations in cephalopod prey at each stranding location is likely confounded by temporal variations in cephalopod distribution, abundance and growth rates resulting from environmental factors, rather than differences in LFPW foraging behaviour. Acknowledging this potential bias, differences were apparent in the cephalopod assemblages between stranding location, but not between sex or age classes. This result corresponds with LFPW strong socially cohesive groups, with males and females of different age classes commonly foraging together [24].

### Size of cephalopods

Based on the reconstructed size, biomass and known morphometric growth curves of the cephalopods recovered, LFPWs occurring in Tasmanian waters appear to primarily target the adult stages of the majority of cephalopod species consumed. This may indicate foraging preferences for adult-sized cephalopods, related to increasing foraging efficiency. These results support the results of Gales et al. [4], which found a potential selection for larger, mature cephalopods. It is proposed that toothed whales preferentially consume larger, less abundant prey over smaller, more abundant ones [65], with the quality of prey, rather than quantity being a major determinant of foraging strategies required to meet specific energetic requirements [66].

### Correlation between LFPW body size and the three most important cephalopod prey

Previous studies have shown that the size of prey consumed can be dependent on the sex and size of the predator, where larger predators often consume larger prey [67]. Contrary to these findings, this study found that for female LFPWs, and the Marion Bay stranding, larger LFPWs were consuming smaller Ommastrephidae spp. than smaller LFPWs. No correlation was found in LFPWs stranded at the other two locations. There were no correlations between LFPW body size and the size of *L*. *lorigera* or *A*. *lesueuri* consumed. It is acknowledged that many missing values and small sample size may have confounded these results, highlighting the importance of recording total length (as a minimum external measure) for all stranded specimens, when possible [45].

### Ecological characteristics of major prey species

The results from this study show that LFPWs utilising Tasmanian waters are feeding on a diverse range of cephalopods, all of which have complex life cycles, behaviour and habitat requirements.

Although it appears that adult-sized cephalopods are primarily targeted by LFPWs, some subadult and juvenile beak remains were also recovered. The most important cephalopods recovered were: Ommastrephidae sp., *L*. *lorigera* and *A*. *lesueurii*. These species are widespread in epipelagic, mesopelagic and bathypelagic waters, with *L*. *lorigera* and *A*. *lesueurii* commonly found over slopes, seamounts and submarine ridges. Ommastrephids are very widespread and capable of extensive vertical and horizontal migration, while *L*. *lorigera* and *A*. *lesueurii* inhabit lower epipelagic to mesopelagic and bathyal depths during the day and migrate into near surface waters at night.

There are three notable discrepancies between species identified in our study and those recently described by Reid [64]. *Brachioteuthis linkovskyi* was confirmed from our study but is not described by [64]. The *B*. *linkovskyi* beaks identified in this study are identical to the beaks from the type specimen described by Marek Lipinsky, which YC identified. Therefore, it is likely that *B*. *linkovskyi* will be confirmed from Australian waters in the future, with the whole family being in need of revision [64]. Within the family Neoteuthidae, [64] lists *Alluroteuthis antarcticus* as the only Australian representative of Neoteuthidae. However, *Nototeuthis dimegacotyle* is listed for this study. YC described *Nototeuthis dimegacotyle*, which is considered identifiable from *A*. *antarcticus* (mainly an Antarctic species). Similarly, *Onychoteuthis banksii* complex is listed for this study, although [64] lists only *O*. *aequimanus* and *O*. *meridiopacifica*. This family is also in need of further revision.

Few studies have investigated the diving behaviour of LFPWs, however, Northern Hemisphere LFPWs are known to perform deep foraging dives, up to 800 m depth, during foraging periods typically consisting of a series of deep dives and intermittent shallow dives [68–72]. During 1999, Baird et al. [73] conducted tracking studies in the Ligurian Sea off the coast of northwest Italy. Five *G*. *m*. *melas* were tagged for short periods in deep waters (>2000 m), where during the day all five whales spent their time in the top 16 m of the water column. Tags remained attached to two whales after dark, and shortly after sunset both whales made several deep dives (max 360 m and 648 m). It was proposed that these were foraging dives, targeting a time that vertically migrating prey become more readily available as they move closer to the surface. Visser et al. [70] found that LFPWs produce more calls during foraging than non-foraging periods, with increased vocalisations potentially indicative of mediating spacing between group members or synchronisation of foraging activity.

Of the three dietary patterns proposed from previous studies, the Tasmanian stranded LFPWs exhibit a diverse diet, similar to LFPW diet from the Faroe Islands [29, 74], Italy [73], Argentina [41], Northeast Atlantic [31, 32] and the western North Atlantic [30, 75]. This is in contrast to LFPWs stranded in New Zealand waters, which appear to have restricted dietary diversity (≤3 species) dominated by squid [3, 19, 20, 39]: despite the numerous cephalopod species inhabiting New Zealand and surrounding waters. However, sample size for the New Zealand studies were low, many animals had empty stomachs or few dietary remains, and no animals appeared to have eaten in close proximity to the stranding location [3, 20, 39]. Cephalopods were the main prey for two LFPWs from the coast of Normandy (88% numerical proportion), however only two cephalopod species were recovered (*Sepia* sp. and *Sepiola atlantica*) [33]. Five LFPWs taken incidental to fishing operations in the Mid-Atlantic region consumed primarily Atlantic mackerel, *Scomber scombrus* (71%) and long-finned squid, *Loligo pealei* (29%) [34]

One occurrence of red cod remains were recovered from the stomach of a male pilot whale from Maria Island. Since numerous samples of cephalopod tissue were recovered from stomachs from all stranding sites, and the digestion of cephalopod tissue is more rapid than digestion of teleosts [76], our results suggest that LFPWs around the Tasmanian coastline are targeting cephalopods as their primary prey. This lack of preference for fish as prey is also consistent with previous LFPW diet studies around Tasmania and New Zealand [3, 4, 20].

It is acknowledged that the results from this study are confounded by spatial and temporal variation in: (1) stranding events (i.e. each stranding occurred in a different month, a different year, and different location), and (2) cephalopod distribution, movements and life history related to changes in environmental variables [77, 78]. Therefore, any evident differences between the diet of LFPWs from each location may be driven by intra- and inter-annual (and decadal) variability, rather than any real differences. Future diet studies from Tasmanian sites described in this study (i.e. Bicheno, Maria Island, Ocean Beach and Marion Bay) would begin to address some of the variability considerations. Gaps in current knowledge and future cephalopod research priorities should also be addressed, such as linking distribution and abundance to environmental effects on biological processes, and using such knowledge to provide environmental indicators to underpin fishery management [78].

### Potential bias of dietary studies

Although our analysis found minimal remains from non-cephalopod prey items, potential biases of the methods should be considered, including differential digestion of prey items, retention of hard part remains, lack of representation of temporal variability in prey items, and inability to discern primary from secondary digested prey [4, 5, 75, 79, 80].

Other limitations of dietary studies based on stomach content analysis of stranded cetaceans are well known, where the results could be biased towards near-shore prey, perhaps not characteristic of normal foraging behaviour, and by sick whales whose diet does not necessarily represent that of healthy whales; see Pierce and Boyle [81] for a review. Although these limitations likely still apply to mass strandings, they may be minimised since the majority of animals are probably healthy, and still actively foraging prior to death [82, 83].

As a result of the above biases, the estimated importance of particular prey items cannot be guaranteed to reflect that of the true diet of the individuals, or stranded group. However, the analysis of identifiable prey remains confirms the presence of these prey items in the diet of LFPWs, and a relative importance can be estimated. Traditional dietary studies using analysis of stomach contents can also be complemented by novel new techniques such as analysis of faecal DNA [84], tissue lipid profiles [85], fatty acid signature analysis [86], and stable isotope analysis [14, 87, 88] where associated identification of hard part remains from the same individuals may provide a more comprehensive insight into the complete diet of top predators.

The identification of cephalopods using their beaks is a difficult technique, and due to erosion, similarity of beaks, and a lack of taxonomic work on certain families, some species can be easily confused [50]. Comparison of genetic material from identified beaks to known genetic sequences may also be an effective confirmation method to ensure identifications are correct for species known to be easily confused. As an example, for this study most Ommastrephidae spp. were grouped together due to the difficulty to differentiate species, which hindered comparisons of the most important species.

As described above, there are known and well-documented biases that should be considered for diet studies. However, irrespective of the biases, it is clear that cephalopods are an important component of LFPW diet in South Australian waters (i.e. 26 species from 17 families confirmed to be consumed), thus providing a unique insight into a component of the foraging ecology of LFPW.

### Conservation implications

This study shows that cephalopods are the main prey for LFPWs that utilise Tasmanian waters. However, it remains unknown whether LFPWs are migrating through Tasmanian waters, or are resident in nearby offshore waters and occasionally forage inshore, such as during cephalopod inshore migration events described by Desportes and Mouritsen [29]. Recent molecular analysis of worldwide stock structure of LFPWs (i.e. samples from New Zealand, Tasmania and the North Atlantic) showed low haplotype and nucleotide diversity compared to other abundant widespread cetaceans, but strong mtDNA differentiation between ocean basins [89]. Tasmanian samples exhibited the highest diversity at the haplotype and mtDNA level [89]. Of particular significance for this study was the strong differentiation observed among LFPW populations from Tasmanian waters compared to adjacent New Zealand waters [89]. Such strong differentiation was unexpected, because LFPWs are considered widely distributed and nomadic [24, 89]. However, it was suggested that maternal fidelity driven by social organisation or habitat/behavioural specialisation may explain the population structure [89]. These population structure differences may partially explain the apparent differences in diet between the two regions.

As there is no direct catch known for LFPWs in the Southern Hemisphere, the immediate conservation concerns for LFPWS inhabiting Australian waters are a reduction in prey, oceanic pollution and climate change [90, 91]. Effective management of commercial and recreational squid fisheries will assist towards ensuring an adequate prey base for the variety of marine vertebrates (including marine mammals) that rely on cephalopods as a major component of their diet.

## Supporting information

S1 Table Regression equations. (DOCX)

S1 File Correlation between LFPW body size and three most important cephalopod species. (PDF)
