# Supplementary material for: Stomach contents of long-finned pilot whales, Globicephala melas mass-stranded in Tasmania
Source: PLoS One. 2019 Jan 14;14(1):e0206747. doi: 10.1371/journal.pone.0206747 (PMC6331100; doi:10.1371/journal.pone.0206747)
Supplement: S1 Table — (DOCX) [file pone.0206747.s001.docx]

**Supplementary Information 1 –** Regression Equations

| **Family** | **Species** | **Author** | **ML Equation** | **n** | **BM Equation** | **n** |
| --- | --- | --- | --- | --- | --- | --- |
| Ancistrocheiridae | *Ancistrocheirus lesueurii* | Clarke (1980) | ML = 41.3 + 40.75 LRL | 23 | BM = -0.194 + 3.56 In(LRL) | 21 |
| Architeuthidae | *Architeuthis dux* | Clarke (1980) | ML = -55.6 + 59.31(LRL) | 11 | BM = -1.773 + 4.57 In(LRL) | 9 |
| Brachioteuthidae | *Brachioteuthis* sp. | Clarke (1986) | ML = 16.31 + 20.18 LRL | 11 | BM = 0.55 + 1.41 In(LRL) | 11 |
| Chiroteuthidae | *Chiroteuthis* sp. | Clarke (1980) | ML = 11.4 + 24.46(LRL) | 23 | BM = -0.241 + 2.7 In(LRL) | 14 |
| Cranchiidae | *Galiteuthis sp.* | Clarke (1980) | ML = 12.2 + 40.78(LRL) | 39 | BM = 0.728 + 2.34 In(LRL) | 38 |
| Cranchiidae | *Megalocranchia* sp. A | Clarke (1980) | ML = -70.9 + 68.13 (LRL) | 20 | BM = -0.108 + 2.73 In(LRL) | 20 |
| Cranchiidae | *Teuthowenia pellucida* | Lu & Ickeringill (2002) | ML = 22.27 + 29.90 LRL | 72 | BM = 0.71 + 1.94 In(LRL) | 74 |
| Enoploteuthidae | *Enoploteuthis* sp. | Lu & Ickeringill (2002) | ML = -31.46 + 32.73 (LRL) | 13 | BM = -1.99 + 3.92 In(LRL) | 12 |
| Histioteuthidae | *Histioteuthis atlantica* | Horstkotte (2008) | In ML = 3.44 + 0.2594 LRL | 38 | BM = 1.74 + 0.728 LRL | 38 |
| Histioteuthidae | *Histioteuthis macrohista* | Horstkotte (2008) | ML = 10.8 + 12.8 LRL | 52 | In BM = 1.81 + 2.04 In LRL | 52 |
| Histioteuthidae | *Histioteuthis miranda* | Horstkotte (2008) | In ML = 3.02 + 1.15 In LRL | 38 | In BM = 0.878 + 2.96 In LRL | 38 |
| Loliginidae | *Sepioteuthis australis* | Lu & Ickeringill (2002) | ML = -20.78 + 67.89 LRL | 36 | BM = 1.71 + 3.34 In(LRL) | 7 |
| Lycoteuthidae | *Lycoteuthis lorigera* | Lu & Ickeringill (2002) | ML = -13.04 + 34.56 LRL | 45 | BM = 0.32 + 3.00 In(LRL) | 45 |
| Mastigoteuthidae | ?*Mastigoteuthis* sp. A (Clarke) | British Antarctic Survey (unpublished) | ML = 94.424 + 6.203 LRL | 19 | log BM = 0.701 + 1.779 Log LRL | 19 |
| Neoteuthidae | *Nototeuthis dimegacotyle* | no specific equations (Xavier and Cherel 2009) |  |  |  |  |
| Octopoteuthidae | *Octopoteuthis* sp. | Clarke (1986) | ML = -0.4 + 17.33 LRL | 30 | BM = 0.166 + 2.31 In(LRL) | 22 |
| Ommastrephidae | *Martialia hyadesi* | Rodhouse and Yeatman (1990) | ML = 102.0 + 29.47 (LRL) | 67 | BM = 2.405 + 2.012 In(LRL) | 67 |
| Ommastrephidae | Ommastrephidae sp. | Clarke (1986) | ML = 18.53 + 37.44 LRL | nd | BM = 1.11 + 2.64 In(LRL) | nd |
| Onychoteuthidae | *Notonykia africanae* | no specific equations (Xavier and Cherel 2009) |  |  |  |  |
| Onychoteuthidae | *Onykia robsoni* | Lu & Ickeringill 2002 | ML = -652.91 + 151.03 LRL | 6 | BM = -9.15 + 8.07 In(LRL) | 6 |
| Onychoteuthidae | *Onychoteuthis banksii* | Lu & Ickeringill 2002 | ML = 2.31 + 32.75 LRL | 10 | BM = -0.04 + 2.80 InLRL | 10 |
| Pholidoteuthidae | *Pholidoteuthis massyae (based on P. boschmai)* | Clarke (1980) | ML = 11.3 + 41.09 LRL | 12 | BM = 0.976 + 2.83 In(LRL) | 15 |
| Octopodidae | *Octopus maorum (based on Pinnoctopus cordiformis)* | Lu & Ickeringill (2002) | ML = -43.69 + 29.18 LHL | 17 | BM = 2.14 + 2.5 In(LHL) | 12 |
| Ocythoidae | *Ocythoe turberculata* | Lu & Ickeringill (2002) | ML = 2.27 + 5.82 LHL | 16 | BM = -1.05 + 2.51 In(LHL) | 16 |
|  |  |  |  |  |  |  |
| Pseudophycis | *Pseudophycis bachus* | Furlani et al. 2008 | TL = 6.33 OL ^1.62^ mm | 1 |  |  |
