# Supplementary material for: Stomach contents of long-finned pilot whales, Globicephala melas mass-stranded in Tasmania
Source: PLoS One. 2019 Jan 14;14(1):e0206747. doi: 10.1371/journal.pone.0206747 (PMC6331100; doi:10.1371/journal.pone.0206747)
Supplement: S1 File — (PDF) [file pone.0206747.s002.pdf]

Supplementary Information 2 - Correlation between LFPW body size and three most important cephalopod species

Location

Ommastrephidae spp.

Overall, there was a negative correlation between LFPW body size and Ommastrephidae spp. average LRL (i.e. the larger the whale body size, the smaller the prey size: Pearson's correlation coefficient = -0.422: t=-2.324, df=25, p=0.028) (Fig. 1). However, LFPWs in each stranding location were eating different sets of preys, therefore correlations were analysed separately for each location.

Negative correlation was found between LFPW body size and Ommastrephidae spp. average LRL in Marion Bay (Spearman's rank correlation coefficient = -0.72: S=492, p=0.011: Fig. 2) but not in Maria Island (Spearman's rank correlation coefficient = 0.10: S=18, p=0.95: Fig. 3) or Ocean Beach (Spearman's rank correlation coefficient = -0.21: S=200, p=0.554: Fig. 4).

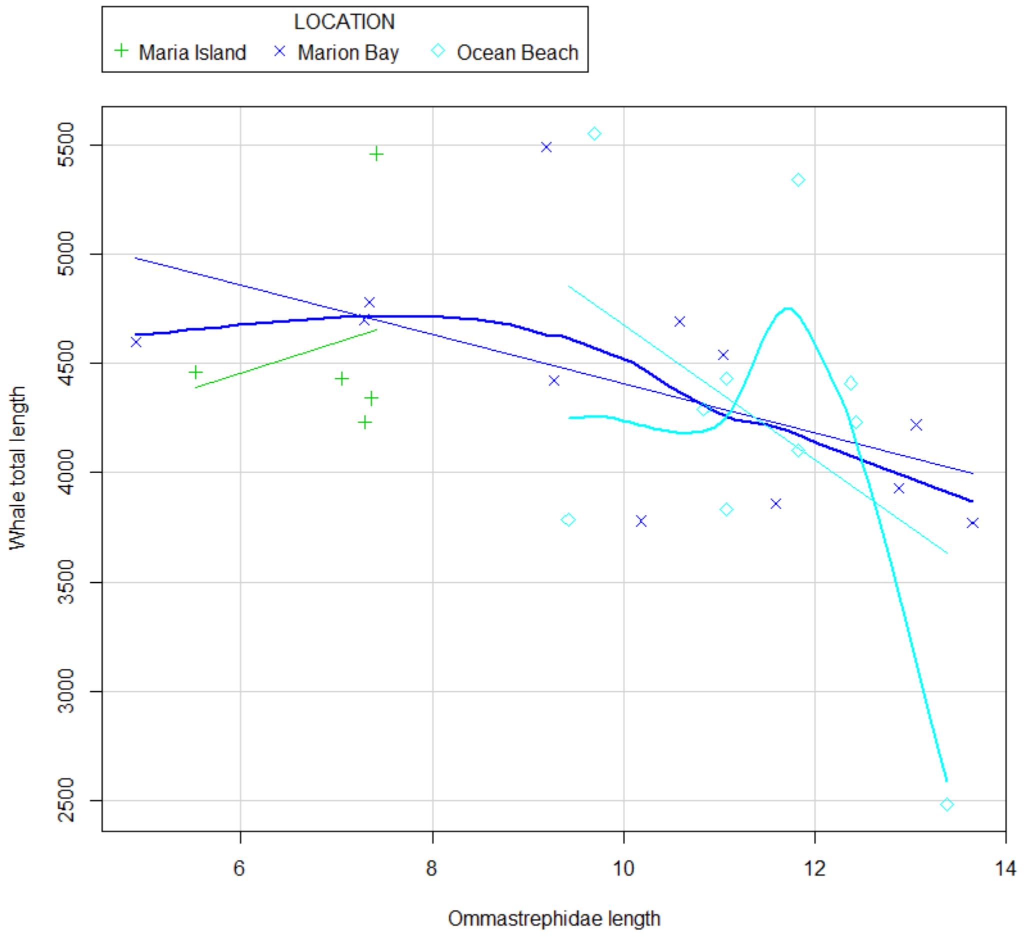

Fig. 1 – Scatterplot of LFPW total length and Ommastrephidae average LRL, stranded at three locations.

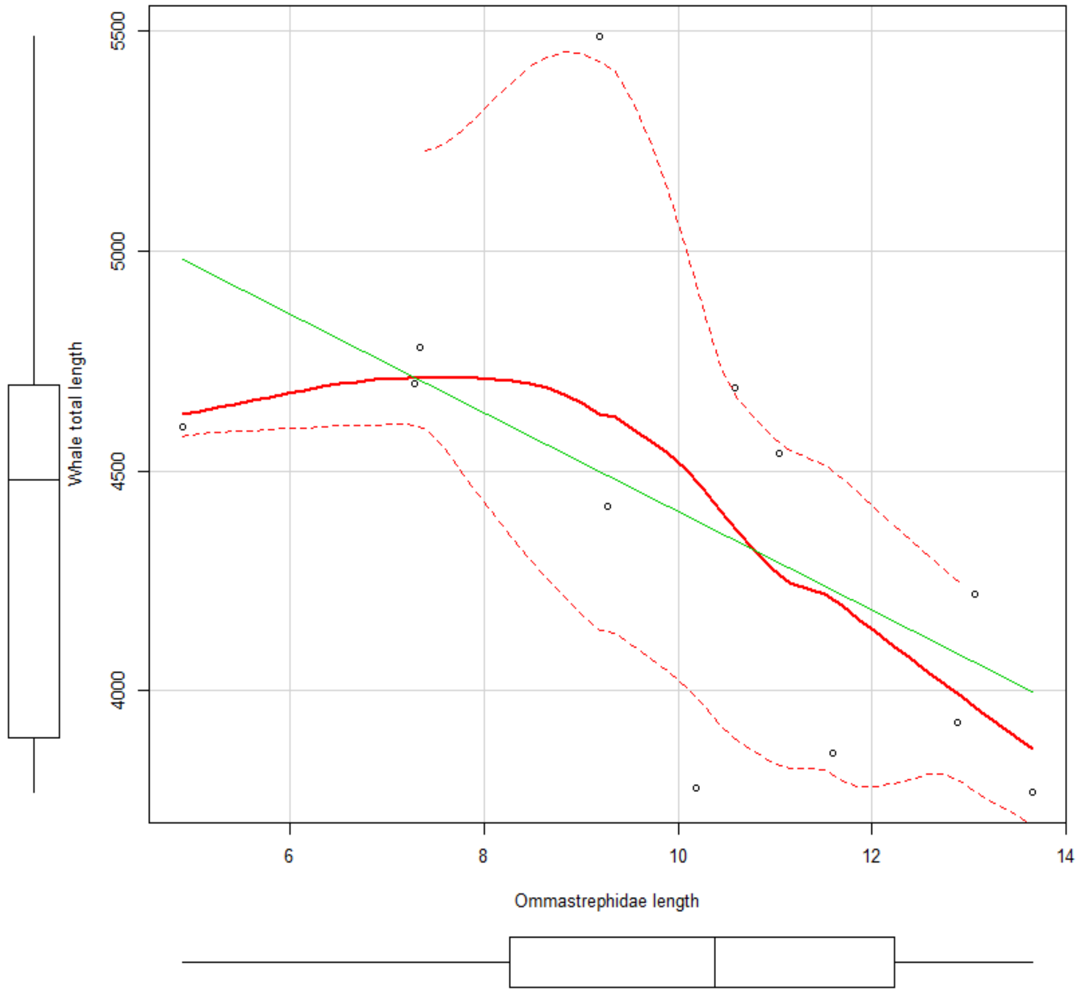

Fig. 2 – Scatterplot of LFPW total length and Ommastrephidae average LRL, stranded at Marion Bay.

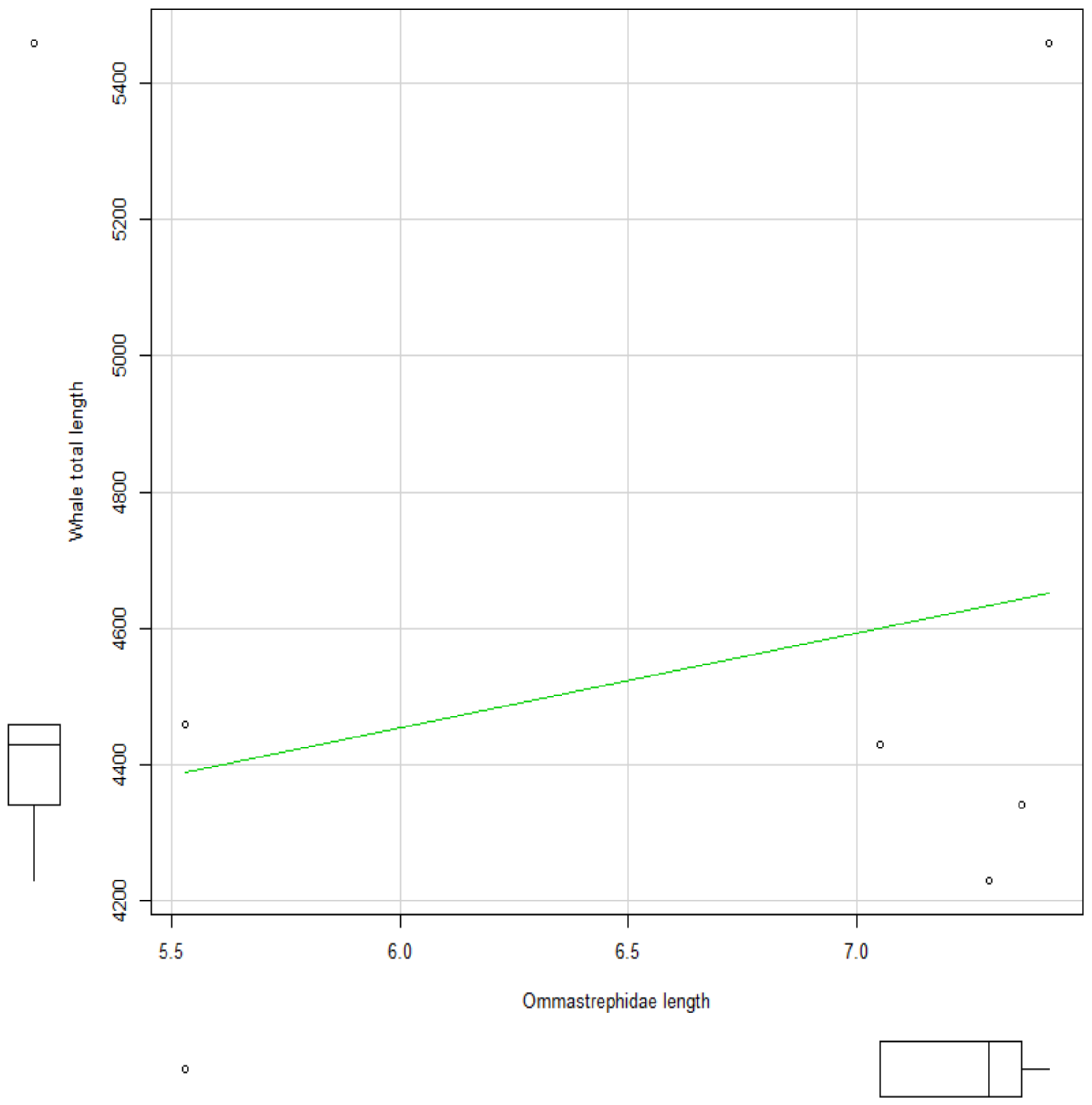

Fig. 3 – Scatterplot of LFPW total length and Ommastrephidae average LRL, stranded at Maria Island.

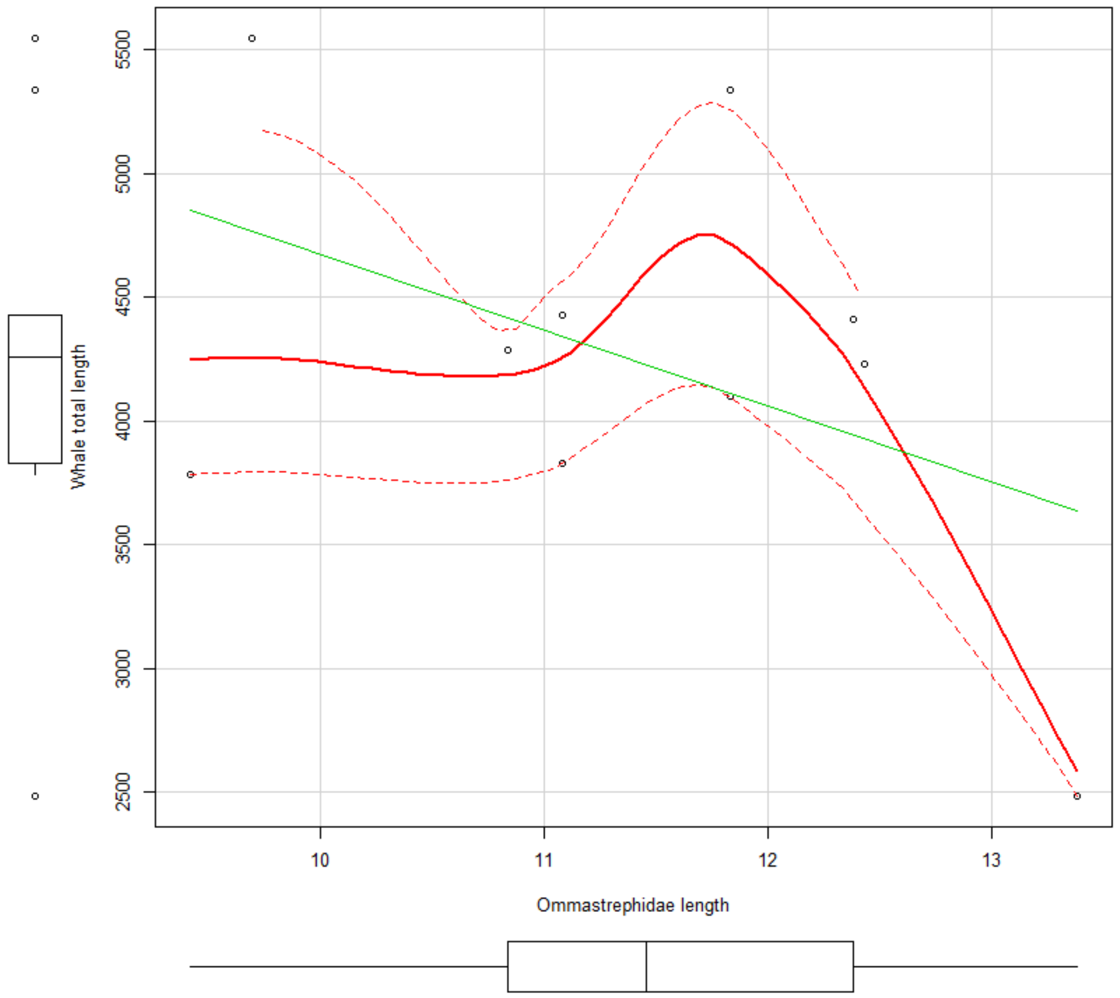

Fig. 4 – Scatterplot of LFPW total length and Ommastrephidae average LRL, stranded at Ocean Beach.

Lycoteuthis lorigera

No correlation was found between LFPW body size and L. lorigera average LRL in Marion Bay (Spearman's rank correlation coefficient = -0.357: S=2747, p=0.09) (Fig. 5). Correlation was not estimated for other locations because Maria Island had only one data point and 2 for Ocean Beach.

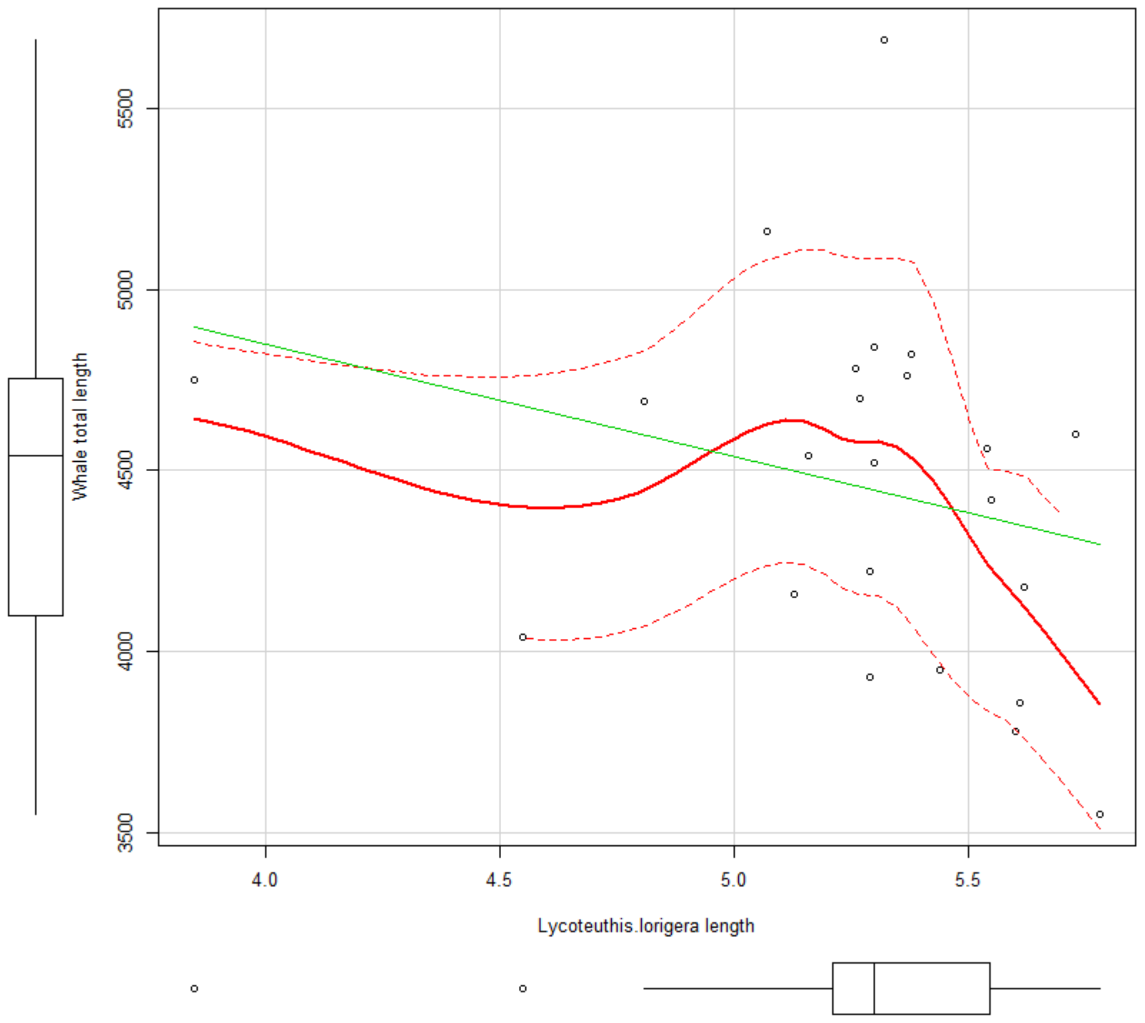

Fig. 5 – Scatterplot of LFPW total length and *L. lorigera* length, stranded at Marion Bay.

Ancistrocheirus lesueuri

No correlation was found between LFPW body size and *A. lesueuri* average LRL in Marion Bay (Spearman's rank correlation coefficient = -0.543: S=54, p=0.297: Fig. 6). Correlation was not estimated for other locations because Maria Island had no data points and only one data point for Ocean Beach.

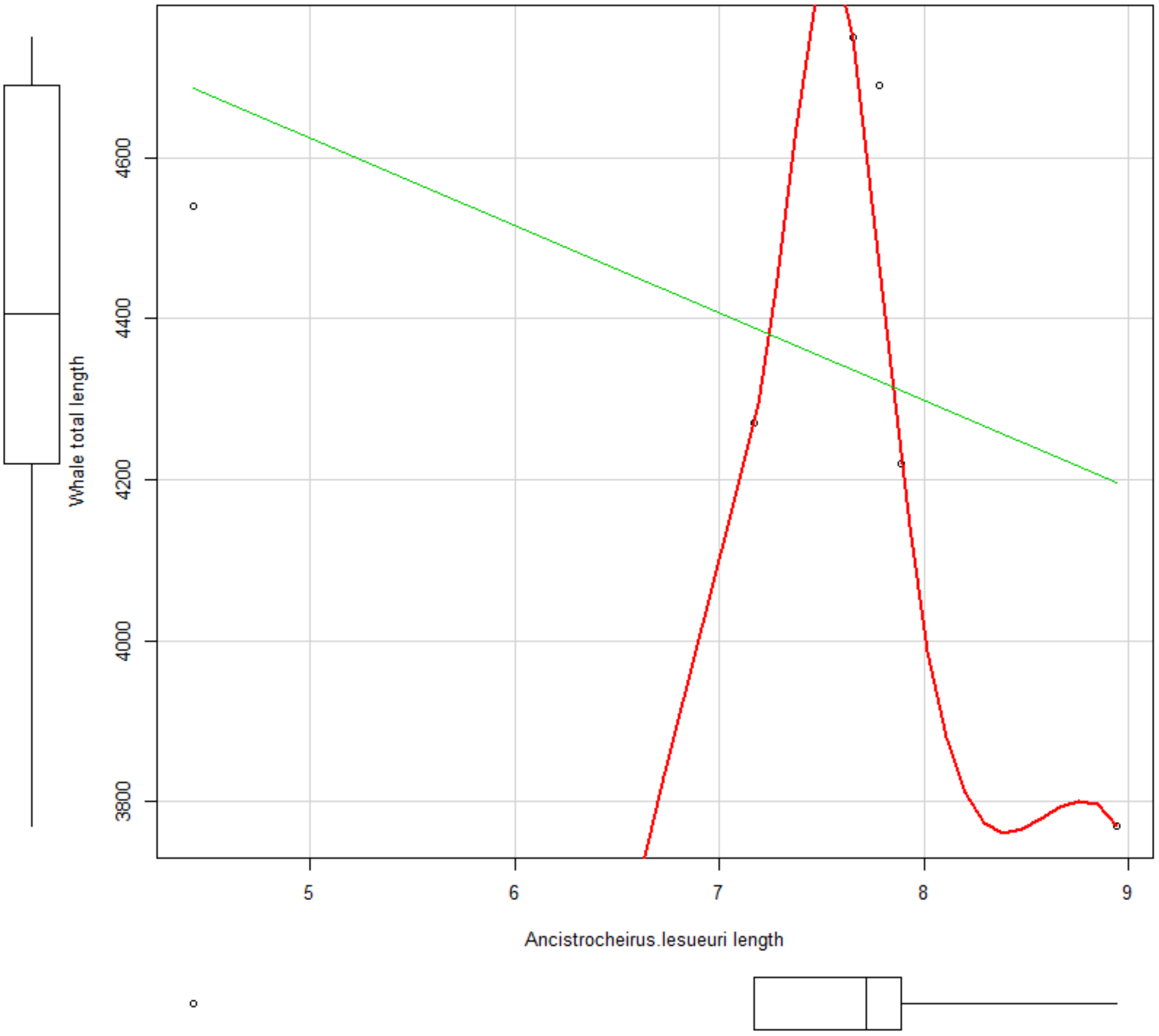

Fig. 6 – Scatterplot of LFPW total length and *A. lesueuri* average LRL, stranded at Marion Bay.

Sex

Ommastrephidae spp.

There was a small negative correlation between LFPW body size and Ommastrephidae spp. average LRL (Fig. 7). For female LFPWs, their body size was negatively correlated with Ommastrephidae spp. average LRL (Spearman's rank correlation coefficient = -0.65: S=1886, p=0.002: Fig 8) but not for male whales (Spearman's rank correlation coefficient = -0.62: S=136, p=0.10) (Fig. 9).

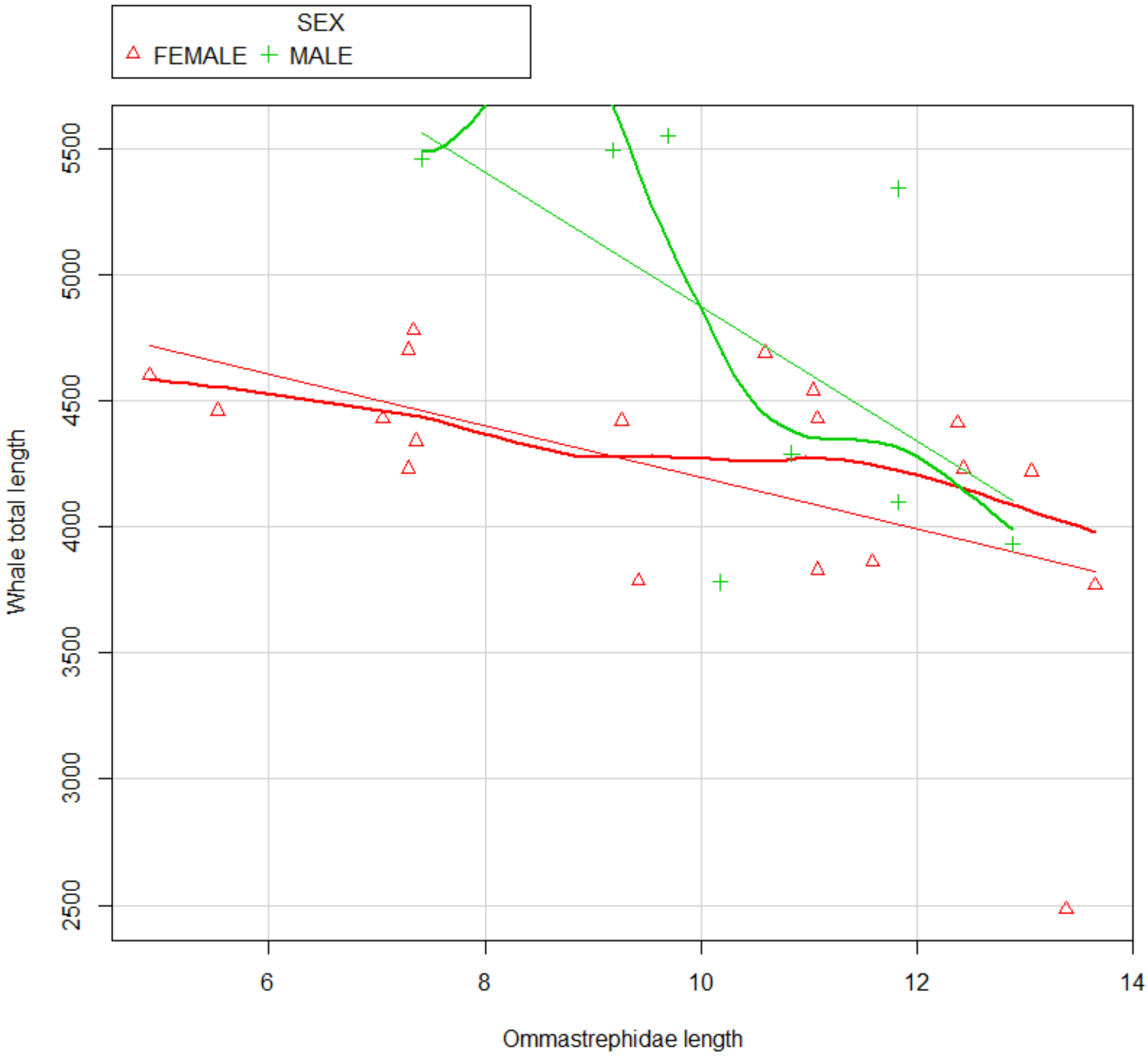

Fig. 7 – Scatterplot of LFPW total length and Ommastrephidae average LRL, for females and males.

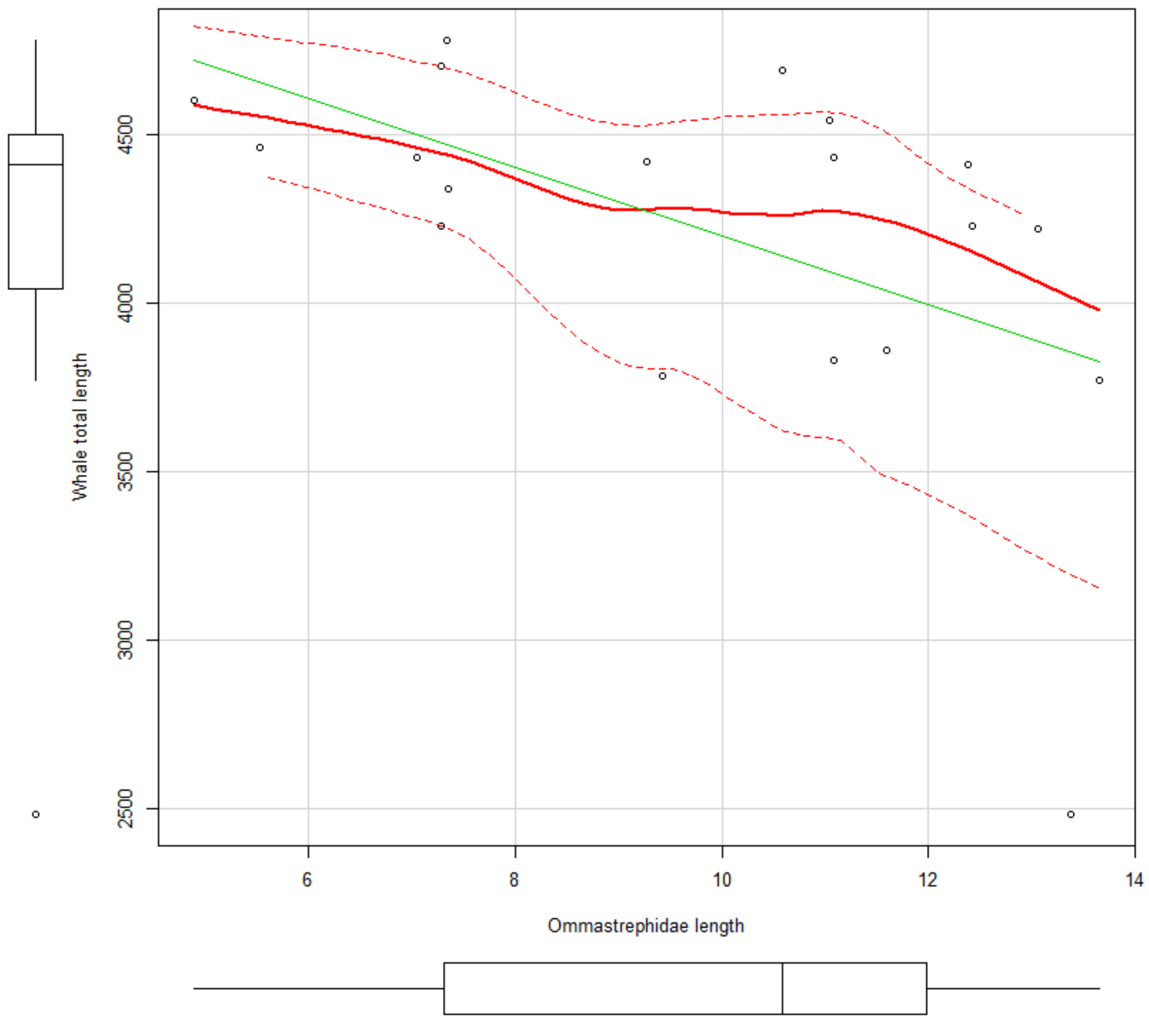

Fig. 8 – Scatterplot of LFPW total length and Ommastrephidae average LRL, for females.

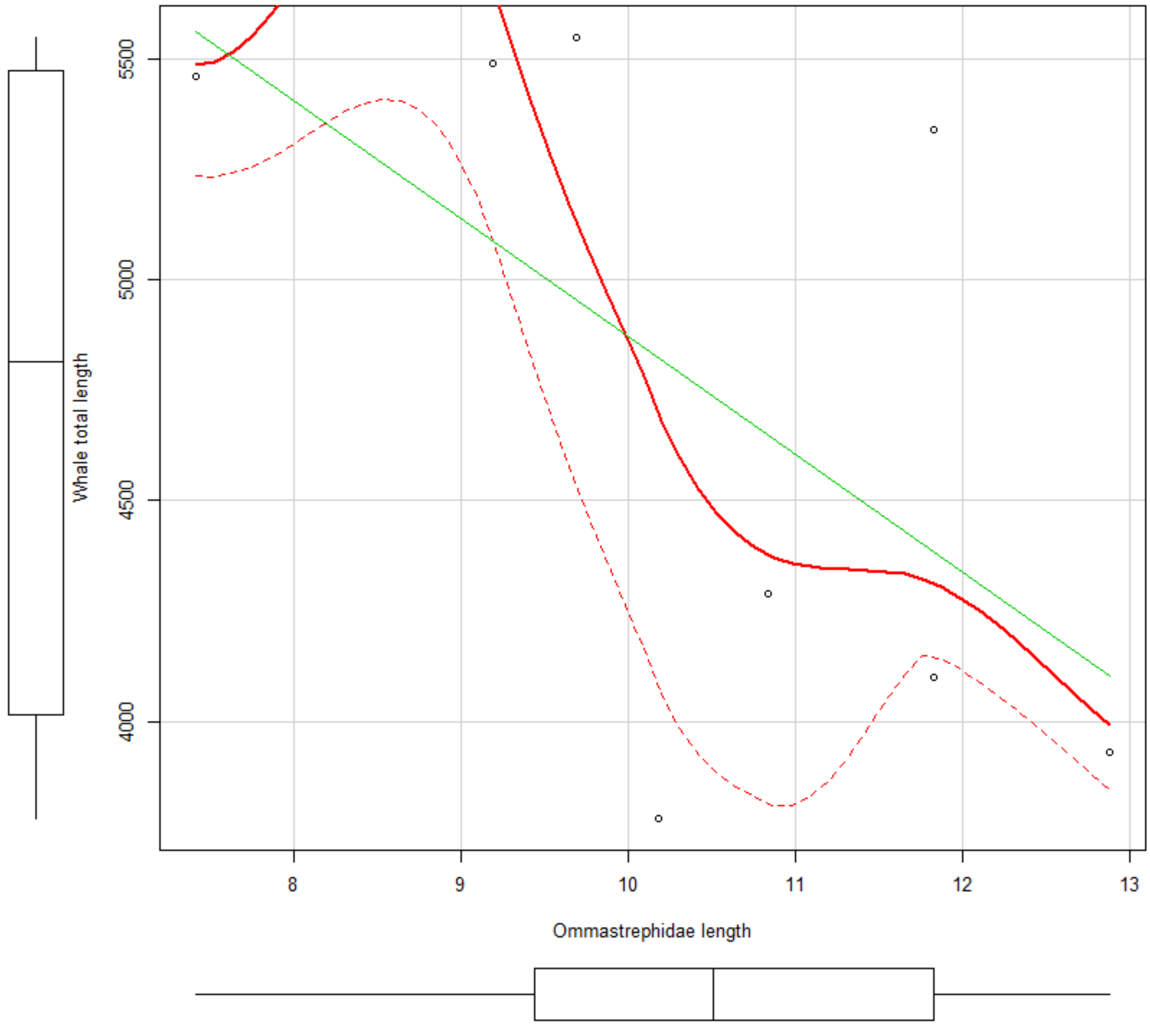

Fig. 9 – Scatterplot of LFPW total length and Ommastrephidae average LRL, for males.

Lycoteuthis lorigera

No correlation between LFPW body size and *L. lorigera* average LRL in both females (Spearman's rank correlation coefficient = -0.208: S=1606, p=0.38: Fig 10) and males (Spearman's rank correlation coefficient = -0.20: S=42, p=0.71: Fig. 11).

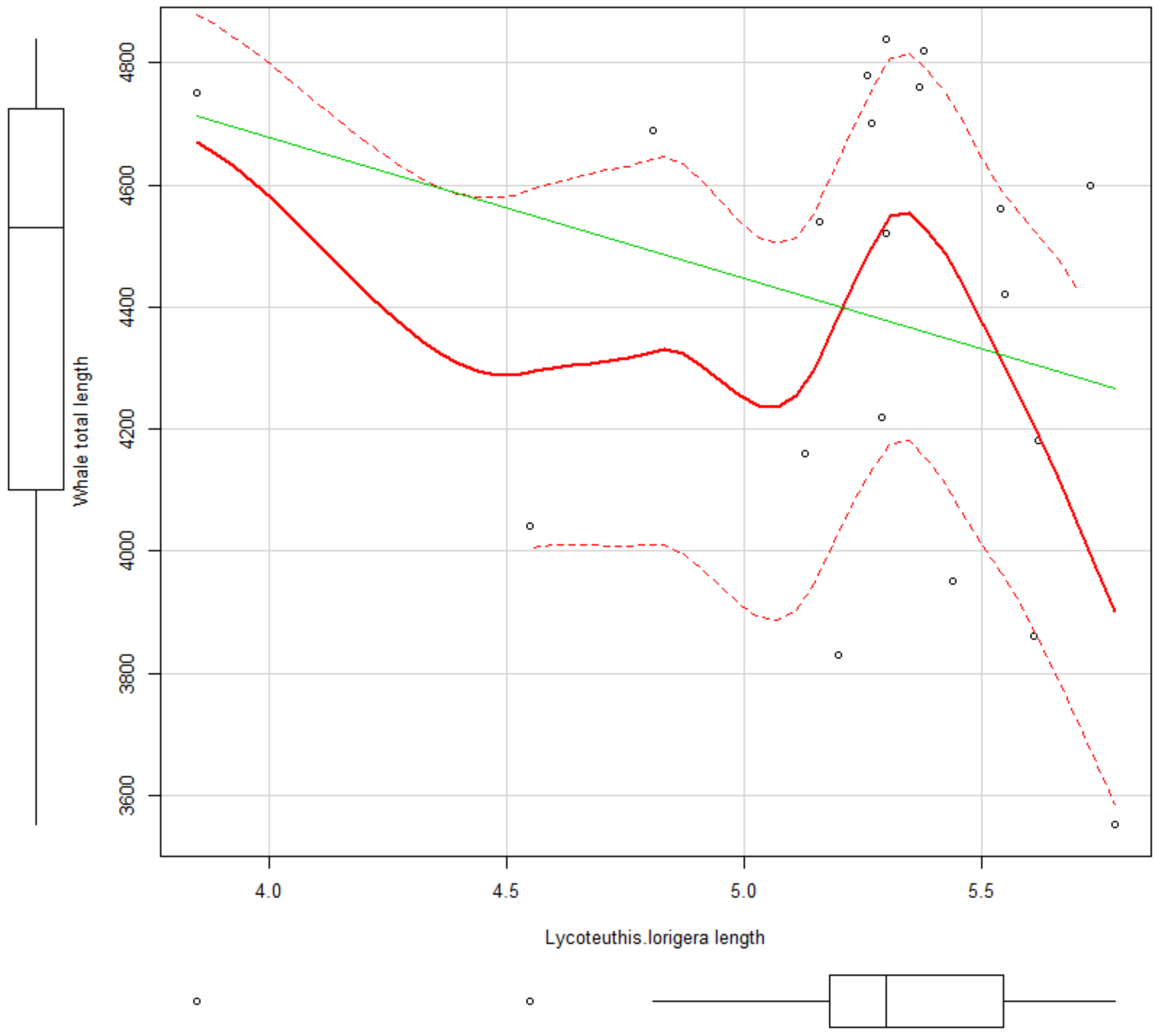

Fig. 10 – Scatterplot of LFPW total length and *L. lorigera* average LRL, for females.

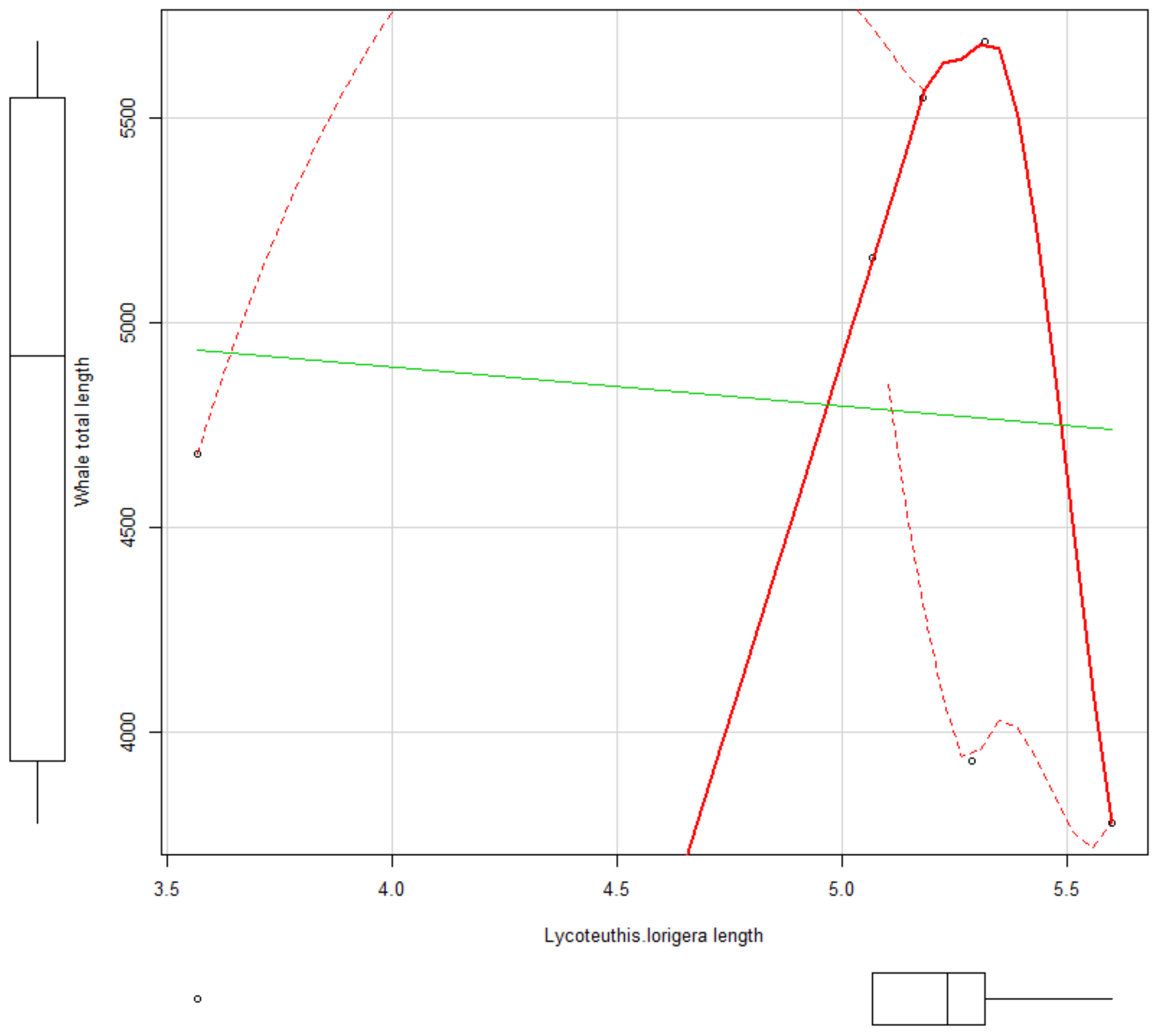

Fig. 11 – Scatterplot of LFPW total length and *L. lorigera* average LRL, for males.

Ancistrocheirus lesueuri

No correlation between LFPW body size and *A. lesueuri* laverage LRL in females (Spearman's rank correlation coefficient = -0.543: S=54, p=0.297: Fig. 12). Correlation was not estimated for males because there was only one data point.

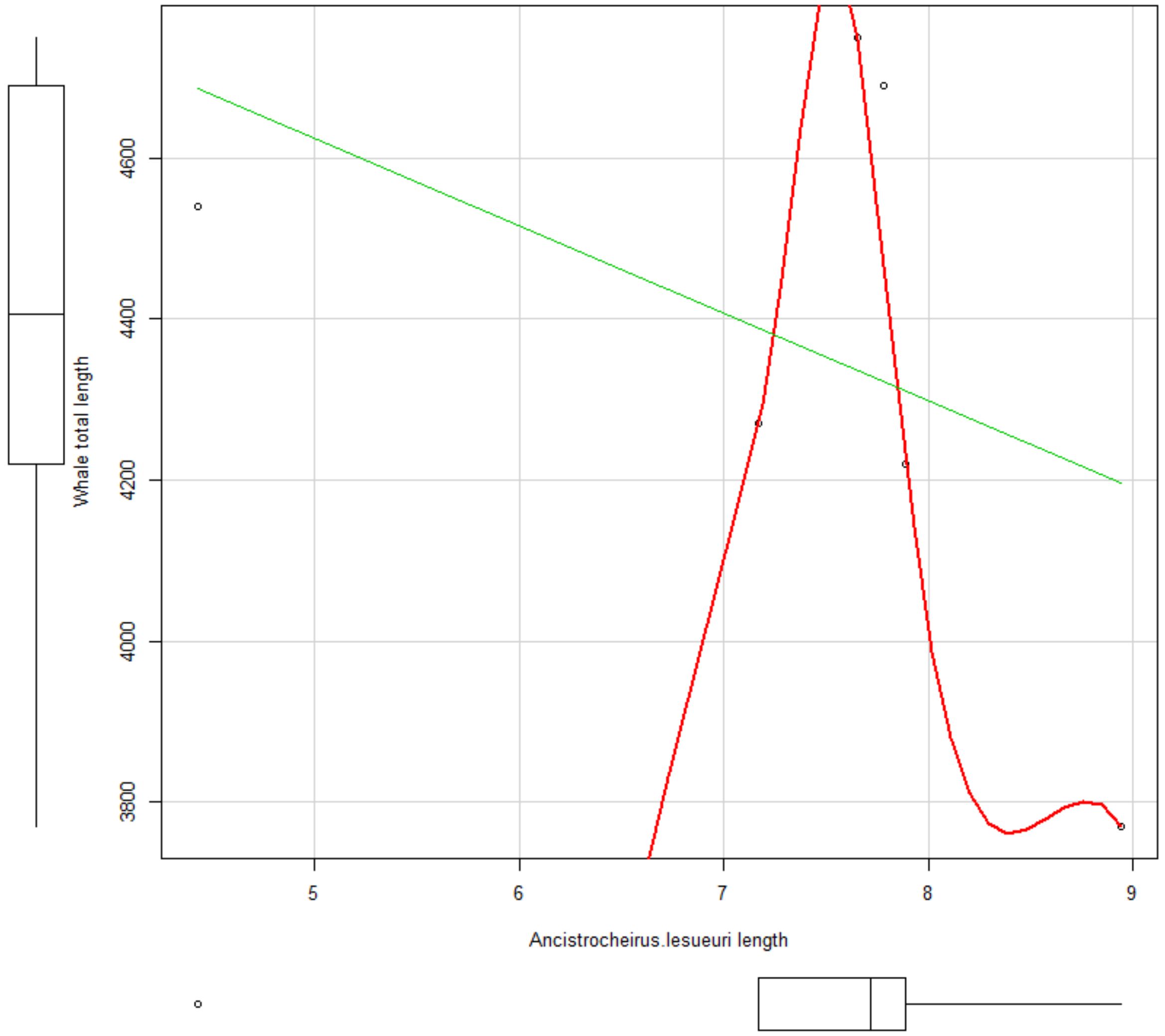

Fig. 12 – Scatterplot of LFPW total length and *A. lesueuri* average LRL, for females.
